# Identification and function analysis of GABA branch three gene families in the cotton related to abiotic stresses

**DOI:** 10.1186/s12870-024-04738-w

**Published:** 2024-01-19

**Authors:** Juyun Zheng, Zeliang Zhang, Nala Zhang, Yajun Liang, Zhaolong Gong, Junhao Wang, Allah Ditta, Zhiwei Sang, Junduo Wang, Xueyuan Li

**Affiliations:** 1 Cash Crops Research Institute of Xinjiang Academy of Agricultural Science ( XAAS ), 830001 Urumqi, Xinjiang P.R. China; 2 Xinjiang Production and Construction Corps, Fifth Division, Eighty-third Regiment, Economic Development Office, 833400 Jinhe, Xinjiang P.R. China; 3 Engineering Research Centre of Cotton, Ministry of Education/College of Agriculture, Xinjiang Agricultural University, 311 Nongda East Road, 830052 Urumqi, Xinjiang P.R. China; 4 Cotton Group, Plant Breeding and Genetics Division, Nuclear Institute for Agriculture and Biology (NIAB) Faisalabad, NIAB-C Pakistan Institute of Engineering and Applied Sciences (PIEAS), Faisalabad, Pakistan

**Keywords:** Gene family, GABA branch, Stress, Expression analysis

## Abstract

γ -aminobutyric acid (GABA) is closely related to the growth, development and stress resistance of plants. Combined with the previous study of GABA to promote the cotton against abiotic stresses, the characteristics and expression patterns of GABA branch gene family laid the foundation for further explaining its role in cotton stress mechanism. Members of GAD, GAB-T and SSADH (three gene families of GABA branch) were identified from the *Gossypium hirsutum*, *Gossypium barbadense*, *Gossypium arboreum* and *Gossypium raimondii* genome. The GABA branch genes were 10 GAD genes, 4 GABA-T genes and 2 SSADH genes. The promoter sequences of genes mainly contains response-related elements such as light, hormone and environment.Phylogenetic analysis shows that GAD indicating that even in the same species, the homologous sequences in the family. The GABA-T gene of each cotton genus was in sum the family had gene loss in the process of dicotyledon evolution. SSADH families *Gossypium hirsutum*, *Gossypium barbadense*, *Gossypium arboreum* and *Gossypium raimondii* were closely related to the dicot plants.GABA gene is involved in the regulation of salt stress and high temperature in *Gossypium hirsutum*.GABA attenuated part of the abiotic stress damage by increasing leaf protective enzyme activity and reducing reactive oxygen species production.This lays the foundation for a thorough analysis of the mechanism of GABA in cotton stress resistance.

## Introduction

As a globally significant economic crop, cotton is primarily used for the production of natural fiber. It is the world’s largest source of textile fiber and an important textile industry raw material. Cotton is a pioneer crop of saline–alkali land and is more resistant to salt and drought than other crops such as wheat and rice. However, abiotic stresses still have a great negative impact on its development and fiber yield (especially seed germination and emergence), which seriously affects the growth, development, and yield of cotton plants. γ-aminobutyric acid (GABA) is a four-carbon nonprotein constituent amino acid which is widely found in almost all eukaryotes and prokaryotes. First discovered in mammals, it acts as a neurotransmitter inhibitor in animals, and has roles in regulating carbon and nitrogen balance, regulating cellular pH, inducing ethylene production, and responding to different stresses, including preventing oxidative stress, inhibiting insect pests, and regulating osmotic pressure and signaling in plants. The GABA-A receptor aluminum-activated malate transporter (ALMT) was identified in wheat and its molecular signaling status was determined. In terms of genetic and physiological effects, GABA has important roles in the stress-response mechanism. Its metabolic pathway includes catalysis of α-ketoglutaric acid by glutamate decarboxylase (GAD) to form GABA, then by GABA transaminase (GABA-T) and succinate half-aldehyde dehydrogenase (SSADH) to form succinate, which is then fed back into the TCA cycle, called the GABA branch (GABA shunt); therefore, this branch is considered to hold a key position in carbon and nitrogen metabolism. It has been shown that the enzymes associated with succinate generation can affect the GABA branch activity, while the downstream enzymes have no effects on GABA metabolism. Therefore, the GABA branch is also one of the carbon replenishment pathways needed to maintain the TCA cycle operation. Because GABA has important roles in plant growth and development, GABA branch genes have been widely studied. As the first key gene in the GABA metabolic reaction, GABA-T was mutated to significantly reduce the salt tolerance in Arabidopsis. Studies in Arabidopsis have found that the POP 2 gene encodes GABA transaminase, which is the primary rate-limiting enzyme in GABA metabolism [1]. Further research has revealed that there are two existing forms of GABA-T in plants [2, 3].

**Fig. 1:**
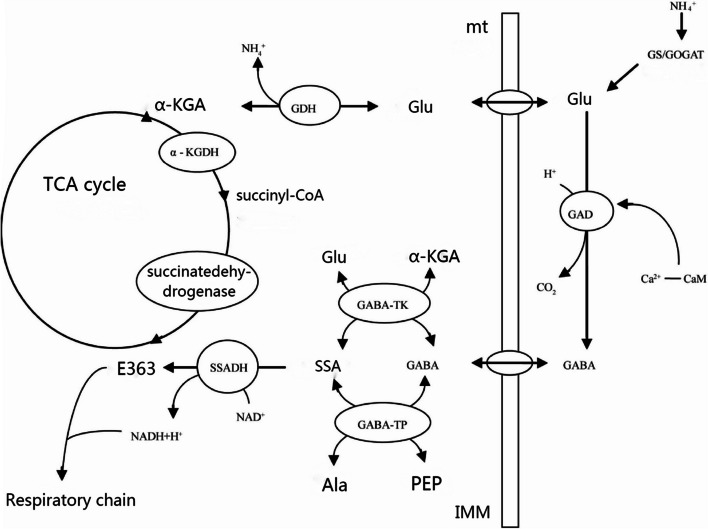
Mechanism diagram of γ-aminobutyric acid

A GABA transaminase with α-ketoglutaric acid, GABA-TP is found in plants in larger quantities than GABA-TK [4]. GABA-T is a PLP-dependent enzyme. In plants, the activity of the GABA transaminase enzyme is the highest when the pH of the plant’s environment is 8.6–9.0. The GABA content increases greatly when plants are subjected to certain stresses, which is mainly regulated by changes in pH in the cytoplasm [5]. GAD genes have been isolated and identified in Arabidopsis, rice, tomato, and other plants, and their expression levels vary among different tissues and developmental periods, so each species shows differences in GABA accumulation. GAD is present in the cytoplasm and has substrate specificity for glutamate [6]. GAD is a complex of pyridoxal phosphate (pyridoxal 5′-phosphate; PLP) and other proteins [7]. The studies of Ling et al. on plants demonstrated that GAD can be activated by Ca_2_/CaM [8]. Further research revealed that the C terminus of GAD has a calmodulin-binding domain (CaM-binding domain; CaMBD) and a pH-sensing site [9]. Bouche et al. found that the Arabidopsis genome contains five GAD orthologs, of which glutamate decarboxylase1 (GADI) is specifically expressed in Arabidopsis roots. Removal of GADI genes prevents the large accumulation of GABA in the root during heat stress, which thus cannot be stimulated during heat stress [3]. In the GABA branch, the last catalytic role belongs to succinate half-aldehyde dehydrogenase (SSADH), which exists in the mitochondria. Mu Xiaomin et al. extracted crude enzyme solutions from radish cotyledons, wheat embryo, and barley seeds and found that the enzymatic activity of SSADH was dependent on NAD^+^ [10]. The SSADH gene has an optimal pH of 9.0–9.5 and has substrate specificity for succinate semialdehyde, using NAD^+^ as a coenzyme in Arabidopsis. Mutation of the SSADH gene in Arabidopsis causes a large accumulation of reactive oxygen species, which has toxic effects on cells (Fig. 1).

Under normal growth conditions, plants have very low GABA levels of about 0.03–2.00 µmol/g (FW) [11]. Higher expression levels of GAD genes in rice and tobacco result in increased accumulation of GABA compared to wild type. Consequently, the stems cannot elongate properly [12]. Meanwhile, knockout of the GAD gene in Arabidopsis causes a reduction in GABA content, which is needed for normal root development [2]. Alterations in the expression level of the POP2 gene in Arabidopsis altered the normal GABA concentration in the stigma, which caused a decrease in the efficiency of ovule fertilization [1]. The GABA content in plants increases considerably and plays a significant role in growth and development under different stress conditions, including acid–base stress [13], freezing [14], thermal stimulation [15], hypoxia [16], disease and pest stress [17], and high salt [18]. Fait et al. proved that GABA metabolism plays an important role in carbon and nitrogen metabolism in plants [4]. GABA is transported into the mitochondria, where it is converted into succinic semialdehyde by GABA transaminases using either α-ketoglutarate (by GABA-TK) or pyruvate (by GABA-TP) as an amino-acid acceptor. Succinic semialdehyde is then reduced by succinic semialdehyde dehydrogenase (SSADH) to form succinate, which enters the tricarboxylic acid (TCA) cycle. Both ATP and NADH can inhibit the activity of the SSADH enzyme. Succinyl-CoA ligase and α-ketoglutarate dehydrogenase (α-KGDH) are two enzymes of the TCA cycle that are bypassed by the GABA shunt and sensitive to oxidative stress. Intermediate products produced during plant growth, such as oxaloacetate and α-ketoglutaric acid, can be used to synthesize other products that help plants to protect themselves against different stress conditions. However, if these intermediates are employed broadly, they will affect the normal operation of the TCA cycle, thus affecting the normal growth and development of the plant [2]. Normally, these intermediates are supplemented by oxaloacetate generated by the PEP pathway, but this pathway does not proceed normally when plants are subjected to certain stress conditions. At this point, it becomes necessary to provide carbon for the GABA cycle [19]. GABA metabolism can function as a molecular negative feedback signal involved in the regulation of stomatal opening, root hair development, and endoreplication in leaf cells. The most recent studies confirm that GABA participates in plant water use efficiency during drought stress through the regulation of stomatal opening.The main focus of this study was the identification of three gene families of the GABA branch (GADs, GABA-Ts, and SSADHs) in the genomes of *G. hirsutum* (upland cotton), *G. barbadense* (sea island cotton), *G. arboreum* (Asian cotton), and *G. raimondii* using bioinformatics analysis. At the cotton seedling stage, phylogenetic analysis was performed under drought conditions, and transcriptome analysis was also performed under heat and salt stress. The expression patterns of the GABA gene family in terms of drought resistance, heat tolerance, and salinity tolerance at the cotton seedling stage were also identified. This lays a foundation for the further study of the role of GABA in regulating cotton stress tolerance mechanisms.

## Experimental materials and methods

### Experimental materials

Four *Gossypium hirsutum* (*G. hirsutum*) varieties were used for investigation of drought and heat stress conditions. Two varieties, Xinluzhong 82 (drought-tolerant) and Kexin 001 (drought-susceptible), were cultivated under drought stress conditions. Two other varieties, Xinluzao36 (heat-tolerant) and Che61-72 (heat-susceptible), were cultivated under heat stress conditions. The materials were provided by the *G. hirsutum* Research Group of the Economic Crop Research Institute of Xinjiang Academy of Agricultural Sciences, China. The experiment used a CRD (completely randomized design) experimental design. The conserved seeds of these four cultivars of cotton were disinfected in 15% hydrogen peroxide solution for 4 h and then rinsed twice with sterile water.

Drought stress experiment: The seeds were grown for three days in Petri dishes. Filter paper was used for germination. Seedlings were transferred to hydroponic conditions and cultivated for 25 days to the third-leaf stage. The water was changed every fourth day, and 1/2 Hoagland nutrient solution was used for hydroponic conditions. Seedlings with consistent growth were selected for treatment. For both drought-tolerant and drought-sensitive varieties, 17% PEG6000 was used for drought treatment at the third-leaf stage, and the leaves were collected at three different time points (0, 12, and 24 h) after treatment.

Heat stress experiment: The seeds were sown in germination boxes (length 12 cm × width 12 cm × height 6 cm) containing 1 kg of sterilized sand and allowed to germinate. In each germination box, 10 seeds were sown at equal intervals, and the sowing depth was 2 cm. After sowing, 300 mL of deionized water was added to each box to saturate the sand with water, then 200 mL water was added to each box every 2 days. The germination boxes were placed in an illumination incubator (LRH250-G, Hangzhou Deju Instrument, Hangzhou, China). The growth conditions of the seedlings were set as follows: photoperiod, 16 h of light and 8 h of darkness; temperature, 28 °C during the day and 20 °C at night. When the cotton seedlings had grown to the third-leaf stage, the temperature of the illumination incubator was adjusted to 40 °C for heat stress treatment. Leaf samples were collected at four different time points (0, 4, 8, and 12 h) after treatment.

The collected samples were frozen in liquid nitrogen and stored at − 80 °C. The collected samples comprised three biological replicates. The RNA prep Pure polysaccharide polyphenol plant total RNA extraction kit (TIANGEN Co., Ltd., Beijing, China) was used to extract total RNA.

### Experimental method

#### Retrieval of three family genes of the GABA branch in cotton local BLAST retrieval of the three family genes of the GABA branch

*G. hirsutum* (UTX), *G. barbadense* (ZJU), *G. arboreum* (CRI), and *G. raimondii* genome data (JGI) were obtained from the CottonFGD database (https://cottonfgd.net/ (accessed on)). The TAIR database (http://www.arabidopsis.org/index (accessed on)) was used to download Arabidopsis GAD, GABA-T, and SSADH data; full-length protein sequences of GABA family members were downloaded, including 6 GAD genes (AT2G02010, AT5G17330, AT2G02000, AT1G65960, AT3G17760, AT3G17720), 1 GABA-T gene (AT3G22200), and 1 SSADH gene (AT1G79440). Bioedit software (http://www.mbio.ncsu.edu/bioedit/page2.html (accessed on)) was employed for trimming and alignment of the protein sequences of the different cotton varieties, including upland, Asian, and *G. raimondii* varieties separately. The GABA branch gene family coding protein sequence of *Arabidopsis thaliana* was used as the query sequence with the E value set to 0.00000000001 and other parameters set to default. The results were exported, and all the gene accession numbers were extracted and saved.

#### Identification of the GABA branch family genes in cotton

The retrieved gene accession numbers were summarized, and the transcript ID of each accession number was edited. TBtools (v0.66836) software (Chenetal., 2018) was employed to extract the full-length protein sequences for each gene. Conserved protein domains of the GABA family genes in cotton (GAD, GABA-T, and SSADG) were identified using Pfam (https://pfam.sanger.ac.uk (accessed on)) (GAD: PF00282, GABA-T: PF00202, SSADH: PF00171), ORF (https://www.ncbi.nlm.nih.Thegov/orffider/ (accessed on)), and the CDD database (http://www.ncbi.nlm.nih. (accessed on)).

#### Information extraction of cotton GABA branch family genes and analysis of physicochemical properties of proteins

The relevant information of cotton GAD, GABA-T, and SSADH genes was obtained from the genome annotation files (UTX, ZJU, CRI, JGI) of upland, sea island, Asian, and *G. raimondii* cotton genomes from the CottonFGD and Cottongen databases, including the accession number, the location of genes on the chromosome, the number of exons, and the average length. Physical and chemical properties (MW and pI) of GAD, GABA-T, and SSADH genes were identified using the ProtParam online tool (http://web.expasy.Og/protparam/ (accessed on)) and CELLO v2.5 online tool (http://cell.life.nctu.edu (accessed on).

#### Gene phylogenetic analysis of cotton GABA branch family

A gene phylogenetic tree of six evolutionary species was selected, including *G. hirsutum*, *G. barbadense*, *G. arboreum*, and *G. raimondii*. Protein sequences of three GABA branch family genes (GABA-T, GAD, and SSADH) from small Lithuanian moss (*Physcomitrella patens*), oil camphor (*Amborella trichopoda*), rice (*Oryza sativa*), soybean (*Glycine max*), Brachypodium (*Brachypodium distachyon*), and hairy fruit poplar (*Populus trichocarpa*) were downloaded from Phytozome (http://www.phytozome.net/ (accessed on)).

The phylogenetic tree was constructed based on the genes of the six evolutionary species. The phylogenetic tree was constructed using the Mega 7 software. Firstly, the multiple-sequence alignment of cotton GAD, GABA-T, and SSADH genes was performed using the ClustalW tool in Mega 7, with the parameters were set to default. After that, a phylogenetic tree was constructed using the Phylogeny tool in Mega 7 software, using maximum likelihood and the bootstrap method, and the repeat value was set to 1000. The phylogenetic tree was visualized using Figtree v1.4.3 software (http://tree.bio.ed.ac.uk/software/figtree/).

#### Cotton GABA branch family gene structure, protein conserved motifs, and promoter analysis

The genetic structure of the GABA branch gene family was analyzed using the online software Gene Structure Display (http://gsds.cbi.pku.edu.cn (accessed on)). Conserved motifs were analyzed using MEME (http://meme-suite.org (accessed on)), and the promoter sequences were identified using the online software PlantCARE (http://biinformatics.psb.ugent.be/webtools/plancare/html/ (accessed on)).

#### Location of Cotton GABA branch genes on the chromosomes

A Perl script in the Linux system was used to extract the length and location information of each chromosome and the GABA branch genes of the four cotton species, namely upland cotton, sea island cotton, Asian cotton, and *G. raimondii*. Genome data annotation files were used to locate the chromosomes of the four cotton species. A distribution plot of the GABA branch gene family on 13/26 chromosomes was constructed using the Map Gene 2 Chromosome v2 online tool (MG2C, http://mg2c.iask.in/mg2c_v2.0/ (accessed on)).

#### GABA branch family and analysis of gene duplication events

The local dataset was constructed using the CDS sequences of the GAD, GABA-T, and SSADH genes. After that, each GABA branch family gene was searched with the BlastN tool (TBtools) as the query sequence in the local dataset built in the previous step. Finally, the Perl script was used to retrieve all gene pairs in the GABA branch gene family. Using the ClustalW v2.1 software (Thompsonetal., 2002), a full sequence alignment of the replicated gene pairs was performed. Then, the KaKs_Calculator 2.0 software (Wangetal., 2010) was utilized to calculate the nonsynonymous substitution rate (Ka) and the synonymous substitution rate (Ks) for these replicated gene pairs, and the environmental selection pressure was further analyzed using the ratio of Ka to Ks.

#### *G. Hirsutum* protein interaction analysis

The GhGABA protein sequence was added to the online server STRING version 11.0 (https://string-db.org (accessed on)) to predict the protein–protein interaction networks.

#### Analysis and treatment of transcriptome data of drought stress and heat stress in *G. Hirsutum*

##### Total RNA quality detection

The total RNA quality was determined via UV absorption and denaturing agarose gel electrophoresis.

##### Illumina and ONT sequencing

Based on sequencing by synthesis (SBS), the Illumina HiSeq high-throughput sequencing platform (drought stress) and PromethION platform (heat stress) were used to sequence the cDNA library and produced large quantities of high-quality raw data.

##### Comparison with reference genome sequences

The genome of *Gossypium hirsutum* was used as a reference for sequence alignment and subsequent analysis. The reference sequence was downloaded from the Cottongen and CottonFGD databases. HISAT2 (Li et al., 2014) was used for the comparison of RNA sequencing experiment reads, as it had high comparison efficiency.

##### Bioinformatics analysis

The raw data were filtered, and high-quality clean data were obtained after removing the spliced sequences and low-quality reads; these clean data were used to perform expression analysis and new gene discovery. Advanced analyses, such as the functional annotation and functional enrichment of differentially expressed genes, were performed according to the expression levels of genes in different samples or groups. Differential expression analysis of two conditions/groups was performed using the DESeq R package (version 1.18.0). The resulting *p* values were adjusted using the false discovery rate (FDR) as described. Differentially expressed genes (DEGs) were screened by DESeq with a threshold value of FDR < 0.01 and foldchange ≥ 2. GO and KEGG functional enrichment of the DEGs was implemented using the GOseq R packages and KOBAS software, respectively. Heatmaps were built using the TBtools (v0.66836) software.

##### qRT-PCR analysis

Differentially expressed GABA branch family genes were verified using qRT-PCR. Primers were designed using the Primer3 software (https://primer3.org) (Table 1), referencing the Tiangen Co., Ltd. (Beijing, China) rapid reverse-transcription kit instructions, and they were amplified using SuperReal PreMix Plus (SYBR Green). qRT-PCR data were analyzed via the 2-ΔΔCt method.

**Table 1:** Primer sequences of differentially expressed genes

Gene ID	**Forward Primer ** **(5′–3′)**	**Reverse Primer ** **(5′–3′)**
Gohir.D01G084900	CCAAAGCAAGACACGCACTGAAAC	GCGAGACAAGTGTGAGGTGAGAATC
Gohir.D11G163100	CCAAAGCAAGACACGCACTGAAAC	CCCAAGTACGAACCCTGCGAATAG
Gohir.A11G156000	CACACTGACTGCCCTCACTATTGG	AGAACTCCTCTTCCGTCTCACCTG
Gohir.A01G100200	CCCTTAGGTAGATCCGAGACTTCCC	GCTGAGTGGAGACAACTGTGATGG
Gohir.A03G113900	ACCGTGCTTCGTGTTGTCATCAG	TTTCTCAGTGGCTAGTCTGGCATTG
Gohir.D01G136200	TTGGCGGAGCGTCTTGTGATTG	CCTCTGGGTTTCAATGGCAGTCTTC
Gohir.A01G144400	TTGGCGGAGCGTCTTGTGATTG	CCTCTGGGTTTCAATGGCAGTCTTC
Gohir.A12G153200	ACCAAGCAACAACAACAGCATTAGC	ACGAGAGGCAAAAGTGGAGTGAA
Gohir.A12G272000	CACAGGTTTGTTGGGAGAAGTTTGC	CCGCTTTCACAGGGTCCATCAC
Gohir.D12G272800	CACAGGTTTGTTGGGAGAAGTTTGC	CCGCTTTCACAGGGTCCATCAC
Actin	ATCCTCCGTCTTGACCTTG	TGTCCGTCAGGCAACTCAT

#### External GABA treatment test and measurement of oxidoreductase activity under drought stress and heat stress

##### Drought stress experiments + GABA

Xinluzhong 82 and Kexin 001 seedlings were selected at 25 d and six hydroponic treatment groups were established for each variety, with three replicates for each group. The following treatments were used: CK: 1/2 intensity Hoagland nutrient solution treatment; experimental group 1: 1/2 intensity Hoagland added to 17% PEG6000; experimental group 2: 1/2 intensity Hoagland with 17% PEG6000 and then 5 mM GABA; experimental group 3: 1/2 intensity Hoagland added to 5 mM GABA; experimental group 4: 1/2 intensity Hoagland added to 0.5 mM vigabatrin (VGB, GABA inhibitor); experimental group 5: 1/2 intensity Hoagland was added to 0.5 mM VGB after the addition of 17% PEG6000. The treated leaves were collected at 0, 12, and 24 h and stored in liquid nitrogen. The SOD, POD, and CAT activity was determined. MDA, H_2_O_2_ content, and the rate of superoxide anion production were also determined.

##### High-temperature stress experiments + GABA

Xinluzao36 and Che61-72 seedlings with consistent growth at 25 d were selected and six sand-culture treatment groups were established for each variety, with three replicates for each group. The following treatments were used: CK: 1/2 intensity Hoagland nutrient solution treatment; experimental group 1: 1/2 intensity Hoagland nutrient solution + 5 mM GABA treatment; experimental group 2: 1/2 intensity Hoagland nutrient solution treatment, and the temperature of the illumination incubator was adjusted to 40 °C for heat stress treatment; experimental group 3: 1/2 intensity Hoagland nutrient solution + 5 mM GABA, and the temperature of the illumination incubator was adjusted to 40 °C for heat stress treatment; experimental group 4: 1/2 intensity Hoagland added to 0.5 mM vigabatrin (VGB, GABA inhibitor); experimental group 5: 1/2 intensity Hoagland was added to 0.5 mM VGB, and the temperature of the illumination incubator was adjusted to 40 °C for heat stress treatment. The treated leaves were collected at 0, 4, 8, and 12 h and stored in liquid nitrogen. The SOD, POD, and CAT activity was determined. MDA, H_2_O_2_ content, and the rate of superoxide anion production were also determined.

#### GABA identification was Applied at the *G. Hirsutum* Seedling Stage

The experiment was carried out from 2019 to 2021 at the 16th Group experimental site of the Xinjiang Academy of Agricultural Sciences (80°50′31″ E, 40°30′13″ N). The five *G. hirsutum* varieties were provided by the Economic Crop Research Institute of Xinjiang Institute of Agricultural Science and Technology (all common local varieties). The experiment was conducted using the submembrane drip irrigation mode with six lines, (66 + 10 cm). Each variety was repeated in three replicates, arranged in random blocks of 50 m, and a theoretical strain of 210,000/hm^2^. Field management was conducted together with field production. GABA was sprayed twice at a concentration of 2 g/L. When spraying 75 L/hm on 11 and 21 April, 100 consecutive plants were randomly surveyed for each variety, and the controls were sprayed with H_2_O.

## Results and analysis

### Characteristics of the GABA branch gene family members

Gene families are groups of genes that derived from a common ancestor which has been duplicated, producing two or more copies. They have obvious similarities in structure and function and encode similar protein products. Thirty GAD family members were identified in the *G. hirsutum* genome (Table 2), *G. barbadense* genome (Table 3), *G. arboreum* genome (Table 4), and *G. raimondii* genome (Table 5) using bioinformatics methods.

**Table 2:** Member genes of the *G. hirsutum* GABA branch family

Name	Gene ID	Amino Acid Length/ aa Length	Amino Acid Molecular Mass/ku Molecular Weight	Isoelectric Point pI	Coding Region Size/bp CDS Size	
GhGAD1	Gohir. A01G144400.1	499	56.80	6.03	1497	Cytoplasmic
GhGAD2	Gohir. A03G113900.1	500	56.69	5.92	1500	Cytoplasmic
GhGAD3	Gohir. A09G172900.1	498	56.51	6.39	1494	Cytoplasmic
GhGAD4	Gohir. A12G153200.1	500	56.65	5.69	1500	Cytoplasmic
GhGAD5	Gohir. A12G272000.1	503	56.38	6.70	1509	Cytoplasmic
GhGAD6	Gohir. D01G136200.1	499	56.70	5.77	1497	Cytoplasmic
GhGAD7	Gohir. D02G138300.1	500	56.67	5.92	1500	Cytoplasmic
GhGAD8	Gohir. D09G168600.1	479	54.32	6.16	1437	Cytoplasmic
GhGAD9	Gohir. D12G156600.1	500	56.62	5.87	1500	Cytoplasmic
GhGAD10	Gohir. D12G272800.1	503	56.36	6.01	1509	Cytoplasmic
GhGABA-T1	Gohir. A08G051200.1	503	55.58	8.70	1509	Periplasmic
GhGABA-T2	Gohir. A11G156000.1	516	56.92	7.63	1548	Periplasmic
GhGABA-T3	Gohir. D08G060900.1	503	55.51	8.93	1509	Periplasmic
GhGABA-T4	Gohir. D11G163100.1	516	56.87	8.02	1548	Periplasmic
GhSSADH1	Gohir. A01G100200.1	520	55.76	8.06	1560	Cytoplasmic
GhSSADH2	Gohir. D01G084900.1	520	55.75	8.53	1560	Cytoplasmic

**Table 3:** Member genes of the *G. barbadense* GABA branch family

Name	Gene ID	Amino Acid Length/aa Length	Amino Acid Molecular Mass/ku Molecular Weight	Isoelectric Point pI	Coding Region Size/bp CDS Size	
GbGAD1	GB_A01G1592	498	56.80	6.03	1497	Cytoplasmic
GbGAD2	GB_A03G1393	499	56.79	6.03	1500	Cytoplasmic
GbGAD3	GB_A09G2004	478	54.23	6.05	1437	Cytoplasmic
GbGAD4	GB_A12G1803	499	56.65	5.69	1500	Cytoplasmic
GbGAD5	GB_A12G3025	502	56.44	7.05	1509	Cytoplasmic
GbGAD6	GB_D01G1694	498	56.66	5.77	1497	Cytoplasmic
GbGAD7	GB_D02G1571	499	56.67	6.03	1500	Cytoplasmic
GbGAD8	GB_D09G1851	503	57.32	6.23	1512	Cytoplasmic
GbGAD9	GB_D12G1784	499	56.57	5.97	1500	Cytoplasmic
GbGAD10	GB_D12G3034	320	35.94	5.11	963	Periplasmic
GbGABA-T1	GB_A08G0569	467	51.70	8.52	1404	Periplasmic
GbGABA-T2	GB_A11G1637	515	56.92	8.02	1548	Periplasmic
GbGABA-T3	GB_D08G0584	493	54.79	8.75	1482	Periplasmic
GbGABA-T4	GB_D11G1688	515	56.89	8.02	1548	Periplasmic
GbSSADH1	GB_A01G0969	519	55.79	7.56	1560	Cytoplasmic
GbSSADH2	GB_D01G1038	519	55.75	8.53	1560	Cytoplasmic

**Table 4:** Member genes of the *G. arboreum* GABA branch family

Name	Gene ID	Length/aa	Molecular Weight/ku	pI	CDS Size/bp	Subcellular Localization
GaGAD1	evm.model.Ga01G1666	498	56.78	6.03	1497	Periplasmic
GaGAD2	evm.model.Ga03G1550	519	58.85	6.03	1560	Periplasmic
GaGAD3	evm.model.Ga09G1877	478	54.24	6.16	1437	Cytoplasmic
GaGAD4	evm.model.Ga12G0053	502	56.39	7.05	1509	Cytoplasmic
GaGAD5	evm.model.Ga12G1315	474	53.50	5.45	1425	Cytoplasmic
GaGABA-T1	evm.model.Ga08G0600	521	57.74	8.85	1566	Cytoplasmic
GaGABA-T2	evm.model.Ga11G2370	525	57.95	8.02	1578	Cytoplasmic
GaSSADH1	evm.model.Ga01G1070	530	57.26	8.52	1593	Cytoplasmic

**Table 5:** Member genes of the GABA branch family of *G. raimondii*

Name	Gene ID	Amino Acid Length/aa Length	Amino Acid Molecular Mass/ku Molecular Weight	Isoelectric Point pI	Coding Region Size/bp CDS Size	
GrGAD1	Gorai.004G063100.1	503	55.56	8.61	1509	Periplasmic
GrGAD2	Gorai.006G190200.1	479	54.38	6.06	1437	Periplasmic
GrGAD3	Gorai.007G173800.1	516	56.87	8.02	1548	Cytoplasmic
GrGAD4	Gorai.008G169300.1	500	56.62	5.87	1500	Cytoplasmic
GrGAD5	Gorai.008G293200.1	503	56.45	6.25	1509	Cytoplasmic
GrGABA-T1	Gorai.002G169900.1	499	56.70	5.77	1497	Cytoplasmic
GrGABA-T2	Gorai.005G154700.1	546	61.68	6.14	1638	Cytoplasmic
GrSSADH1	Gorai.002G110500.2	520	55.79	8.06	1560	Cytoplasmic

#### Characteristics of GABA branch gene family members in *G. Hirsutum*

**Fig. 2:**
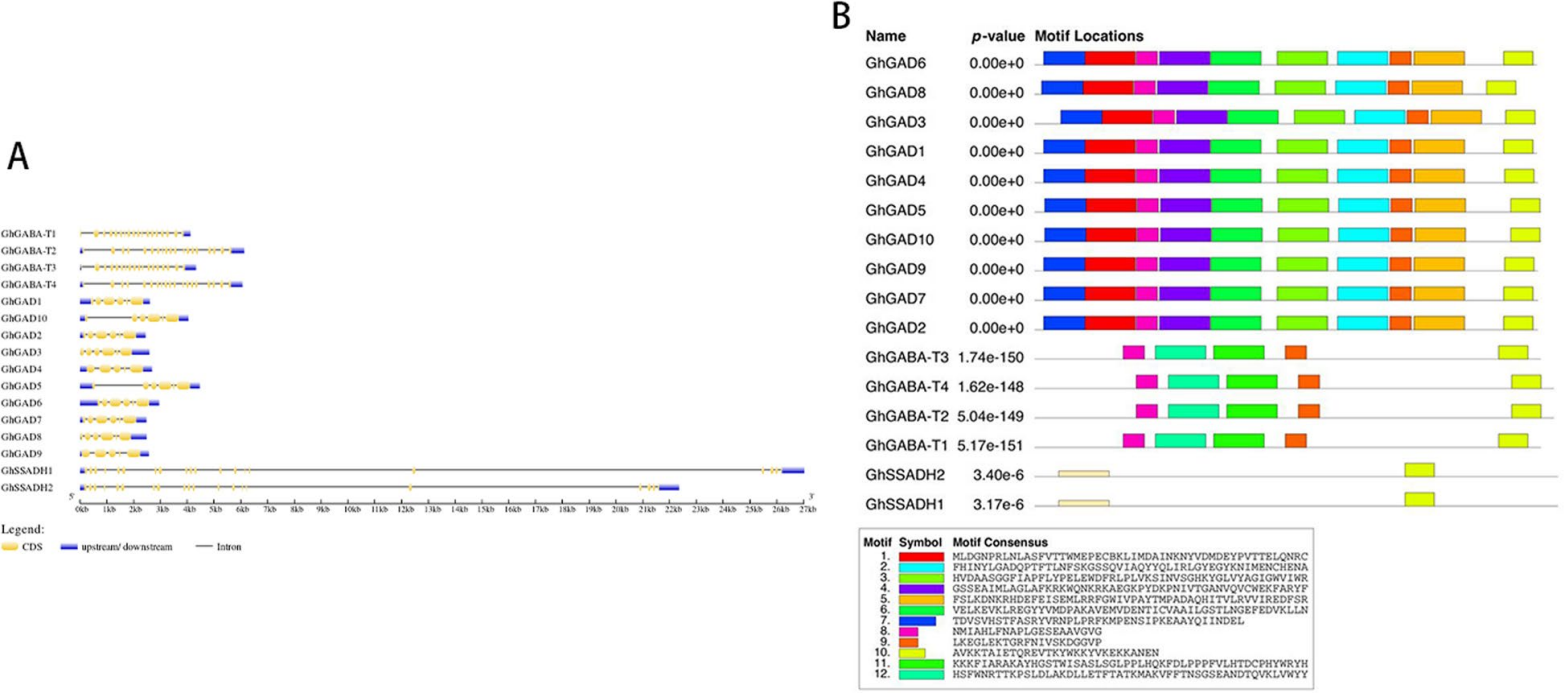
Gene structure and motif analysis of three GABA branch gene family members of *G. hirsutum*. **A** Gene structure (yellow for exons, black lines for introns, blue for upstream and downstream noncoding regions; (**B**) conserved motif distribution as well as conserved motif sequences

The length of the 10-member coding region (CDS) of the GhGAD gene was 1439–1509 bp, the length of the amino acid sequence of the protein was 479–503 aa (number of amino acids), the molecular mass was 54.32–56.80 ku, and the isoelectric point was 5.77–6.70. The four members of the GhGABA-T gene family had a CDS length of 1509–1548 bp, a protein amino acid sequence length of 503–516 aa, a molecular mass of 55.51–56.92 ku, and an isoelectric point of 7.63–8.93. The CDS length of the two members of the GhSSADH gene family was 1560 bp, and the protein amino acid sequence length was 520 aa, with molecular masses of 55.76 and 55.75 ku and isoelectric points of 8.06 and 8.53. Using the WoLF PSORT website to predict *G. hirsutum* GABA, the GhGAD and GhSSADH family member genes were localized to the cytoplasm and the GhGABA-T family member genes to the periplasm (Fig. 2).

Structural analysis of *G. hirsutum* GhGAD family members revealed six exons in GhGAD1,2,3,5,6,7,18,9,10 and five exons in GhGAD4. GhGABA-T family members GhGABA-T1 and -T3 had 18 exons, and GhGABA-T2 and -T4 had 19 exons. Both GhSSADH family members contained 19 exons (Fig. 2A). Comparative analysis of CDS sequence clusters of these genes revealed that genes located in the same evolutionary clade had similar gene structures, including similar intron and exon lengths and a similar intron phase. However, the length difference of introns was larger than that of exons (Fig. 2A). The *G. hirsutum* GABA gene contained 12 motifs. Further analysis found that these three families contained equal numbers of motifs (motifs) and that they were arranged in similar positions (Fig. 2B).

#### Characteristics of sea island cotton GABA branch gene family members

**Fig. 3:**
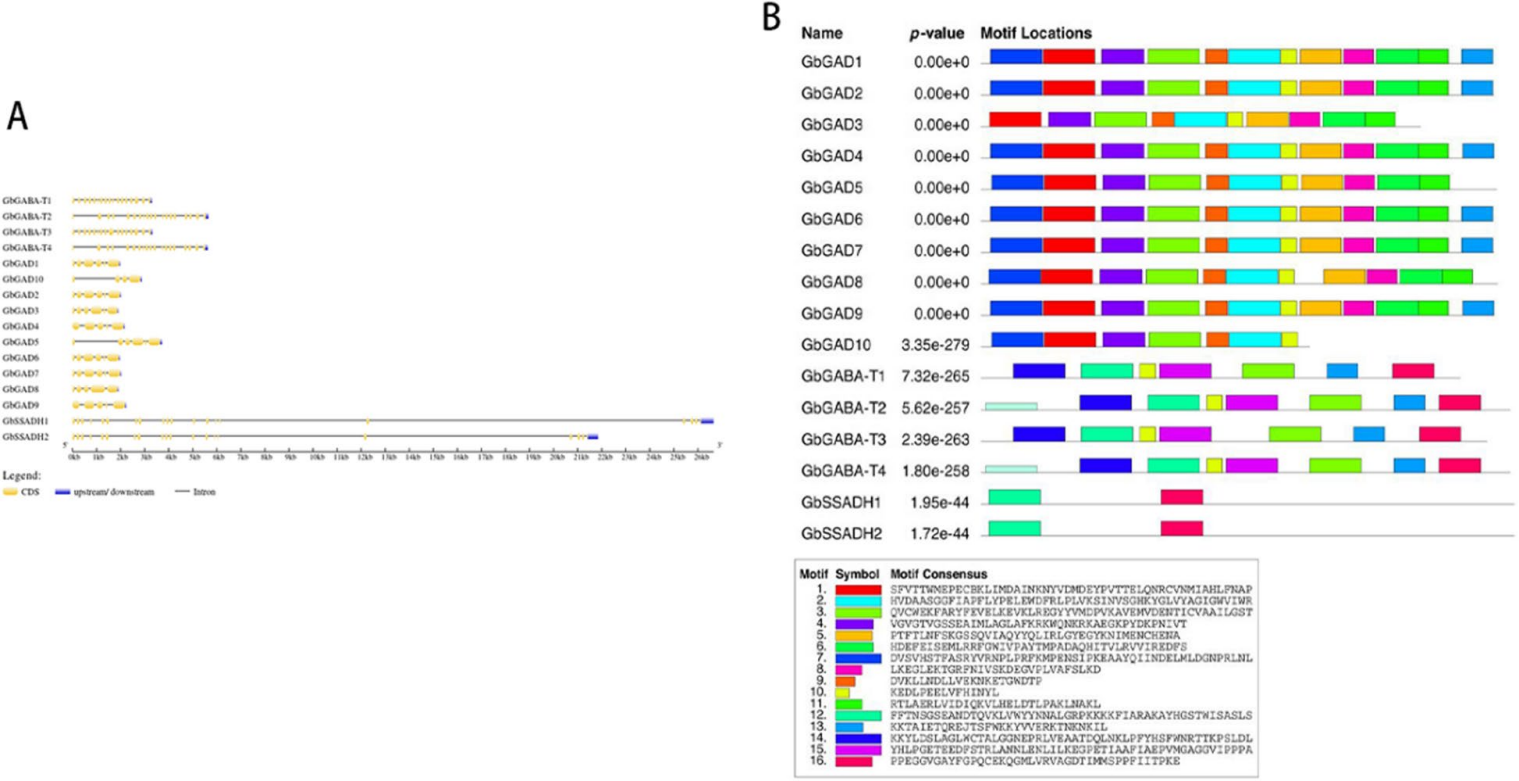
Analysis of GABA branch gene family members of *G. barbadense*. **A** Gene structure (yellow for exons, black lines for introns, blue for upstream and downstream noncoding regions; (**B**) conserved motif distribution as well as conserved motif sequences

The length of the 10-member coding region (CDS) of the GbGAD gene was 963–1512 bp, the length of the protein amino acid sequence was 320–503 aa (number of amino acids), the molecular mass was 35.94–56.80 ku, and the isoelectric point was 5.11–7.05. The four members of the GbGABA-T gene family had CDS lengths of 1404–1548 bp, and the protein amino acid sequence lengths were 467–515 aa, with a molecular mass of 51.70–56.92 ku and an isoelectric point of 7.63–8.93. The CDS length of the two members of the GbSSADH gene family was 1560 bp, with both having protein amino acid lengths of 519 aa, with molecular masses of 55.75 and 55.79 ku and isoelectric points of 7.56 and 8.53. The WoLF PSORT site was used to predict the localization of GABA in the cell, and found that GbGAD10 and GbSSADH family member genes were located in the cytoplasm and the GbGABA-T family members and GbGAD10 genes in the periplasm (Fig. 3).

Structural analysis of the GbGAD family members revealed that all had six exons except GbGAD10, which had four exons. GbGABA-T family members GbGABA-T1 and -T3 had 17 exons, and GbGABA-T2 and -T4 had 19 exons. Both GbSSADH family members contained 20 exons (Fig. 3A). Comparative analysis of CDS sequence clusters of these genes revealed that genes located in the same evolutionary clade had similar gene structures, including similar intron and exon lengths, and a similar intron phase. However, the length difference of introns was relatively large compared with that of exons (Fig. 3A). The *G. barbadense* GABA genes contained 16 motifs, and further analysis found that these three families contained equal numbers of motifs in similar positions (Fig. 3B). GbGAD3 lacked motif 7 and motif 13, and GbGABA-T1 and GbGABA-T3 lacked motif 12.

#### Characteristics of the GABA branch gene family members of asian cotton

The length of the coding region (CDS) of the GaGAD gene of *G. arboreum* was 1425–1560 bp, the length of the amino acid sequence of the protein was 474–519 aa (number of amino acids), the molecular mass was 53.5–55.85 ku, and the isoelectric point was 5.45–7.05. The two members of the GaGABA-T gene family had CDS lengths of 1566 and 1578 bp and the protein amino acid sequence lengths were 521 and 525 aa, with molecular masses of 57.74 and 57.95 ku and isoelectric points of 8.85 and 8.02. One member of the GaSSADH gene family had a CDS length of 1593 bp, a protein amino acid length of 530 aa, a molecular mass of 57.26 ku, and an isoelectric point of 8.52. The WoLF PSORT website was used to predict the localization of *G. arboreum* GABA in cells; GbGAD, except for GbGAD10, and GbSSADH family member genes were located in the cytoplasm and GbGABA-T family members and GbGAD10 were located in the periplasm (Table 4).

**Fig. 4:**
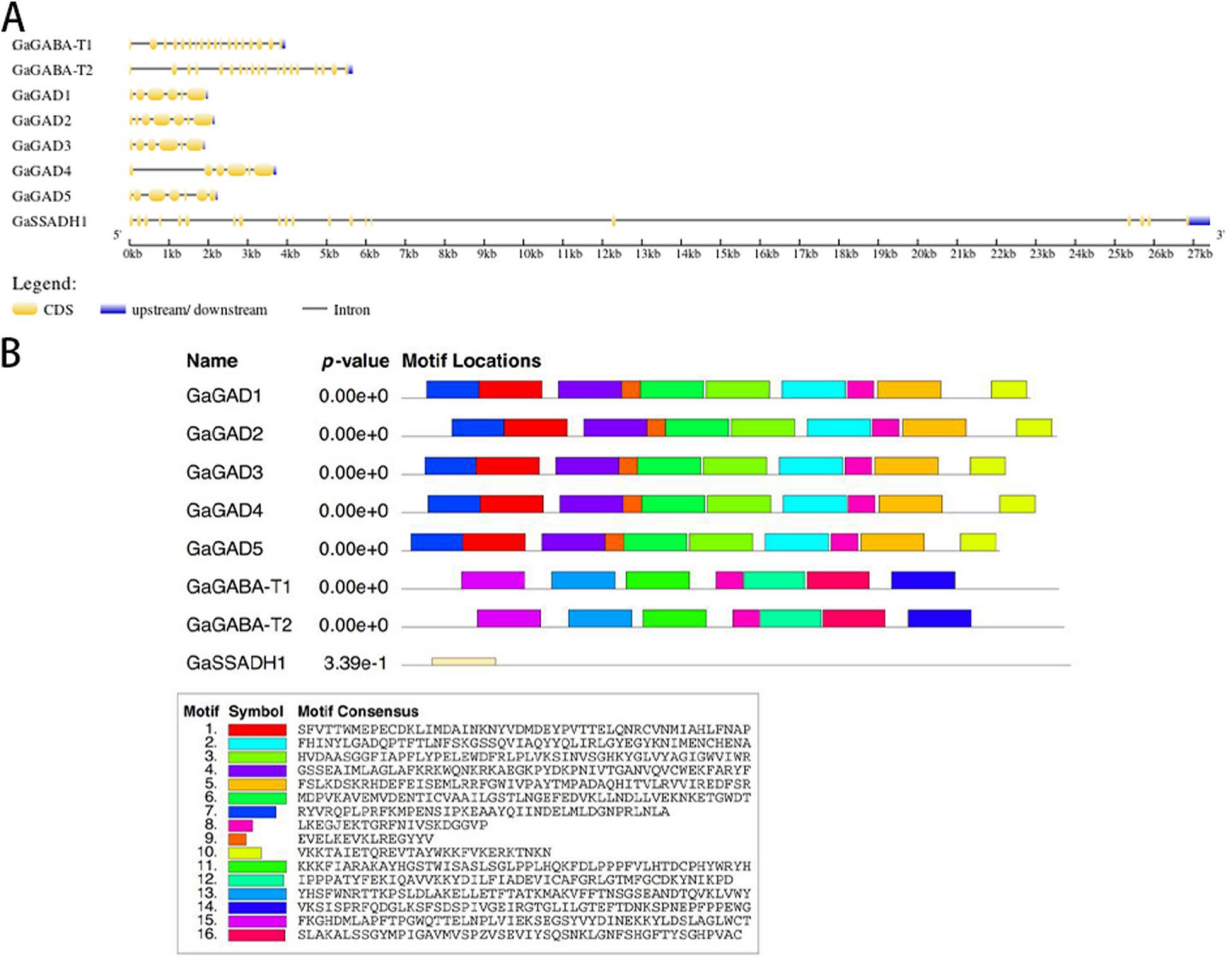
Gene structure and motif analysis of three gene family members of the *G. arboreum* GABA branch. **A** Gene structure (yellow for exons, black lines for introns, blue for upstream and downstream noncoding regions; (**B**) conserved motif distribution as well as conserved motif sequences

Structural analysis of *G. arboreum* GaGAD family members revealed six exons. Two members of the GaGABA-T family, GbGABA-T1 and GbGABA-T2, appeared to have 18 and 19 exons, respectively. One GbSSADH family member had 20 exons (Fig. 4A). Comparative analysis of CDS sequence clusters of these genes revealed that genes located in the same evolutionary clade had similar gene structures, including similar intron and exon lengths, and a similar intron phase. However, the length difference of introns was larger than that of exons (Fig. 4A). The *G. arboreum* GABA gene contained 16 motifs, and further analysis found that these three families contained equal numbers of motifs in similar positions (Fig. 4B).

#### Characteristics of the GABA branch gene family members of raymond’s cotton

The length of the coding region (CDS) of the GrGAD gene was 1497–1548 bp, the length of the amino acid sequence of the protein was 479–516 aa, the molecular mass was 54.38–56.62 ku, and the isoelectric point was 5.87–8.61. The two members of the GrGABA-T gene family had CDS lengths of 1497 and 1638 bp and protein amino acid sequence lengths of 499 and 546 aa, with molecular masses of 56.70 and 61.68 ku and isoelectric points of 5.77 and 6.14. One member of the GrSSADH gene family had a CDS length of 1560 bp, a protein amino acid length of 520 aa, a molecular mass of 55.79 ku, and an isoelectric point of 8.06. The WoLF PSORT website was used to predict the localization of *G. raimondii* GABA in cells. GrGAD, except GrGAD10, and GrSSADH family member genes localized in the cytoplasm and GrGABA-T family members and GrGAD10 localized in the periplasm (Table 5).

**Fig. 5:**
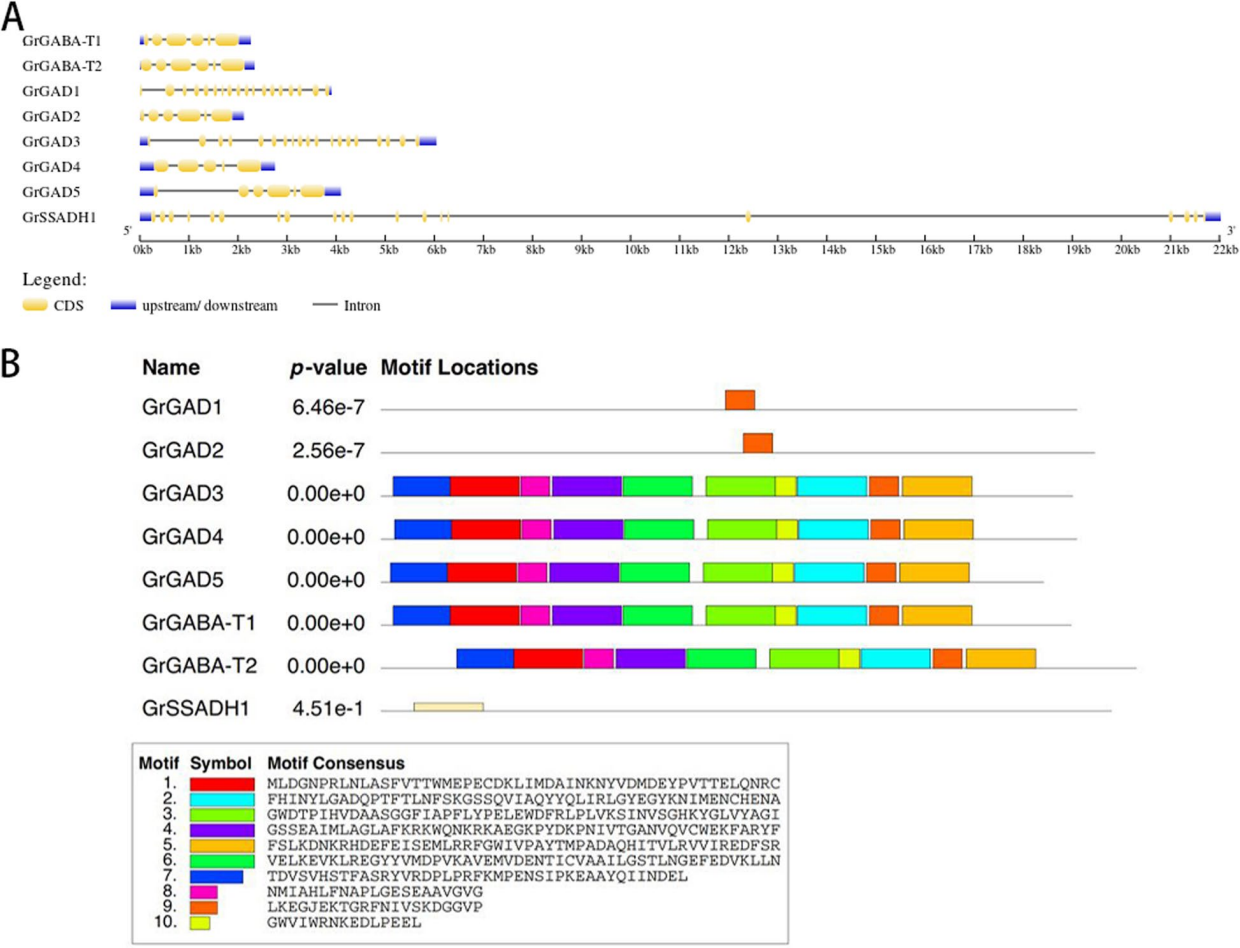
Gene structure and motif analysis of the three gene family members of the *G. raimondii* GABA branch. **A** Gene structure (yellow for exons, black lines for introns, blue for upstream and downstream noncoding regions; (**B**) conserved motif distribution as well as conserved motif sequences

Structural analysis of the *G. raimondii* GrGAD family members identified GrGAD1 with 17 exons; GrGAD3 with 19 exons; and GrGAD2, GrGAD4, and GrGAD6 all containing 6 exons. Both GrGABA-T family members had 6 exons. One GrSSADH family member had 20 exons (Fig. 5A). Comparative analysis of CDS sequence clusters of these genes revealed that genes located in the same evolutionary clade had similar gene structures, including similar intron and exon lengths, and a similar intron phase. However, the length difference of introns was larger than that of exons (Fig. 5A). The *G. raimondii* GABA gene contained 16 motifs. Further analysis showed that the three families contained equal numbers of motifs (motifs) and that they were arranged in similar positions (Fig. 5B). GrGAD1 and GrGAD2 contained only motif 9.

### Promoter and phylogenetic tree analysis of the GABA branch gene family members

#### Promoter analysis of GABA branch gene family members in *G. Hirsutum*

**Fig. 6:**
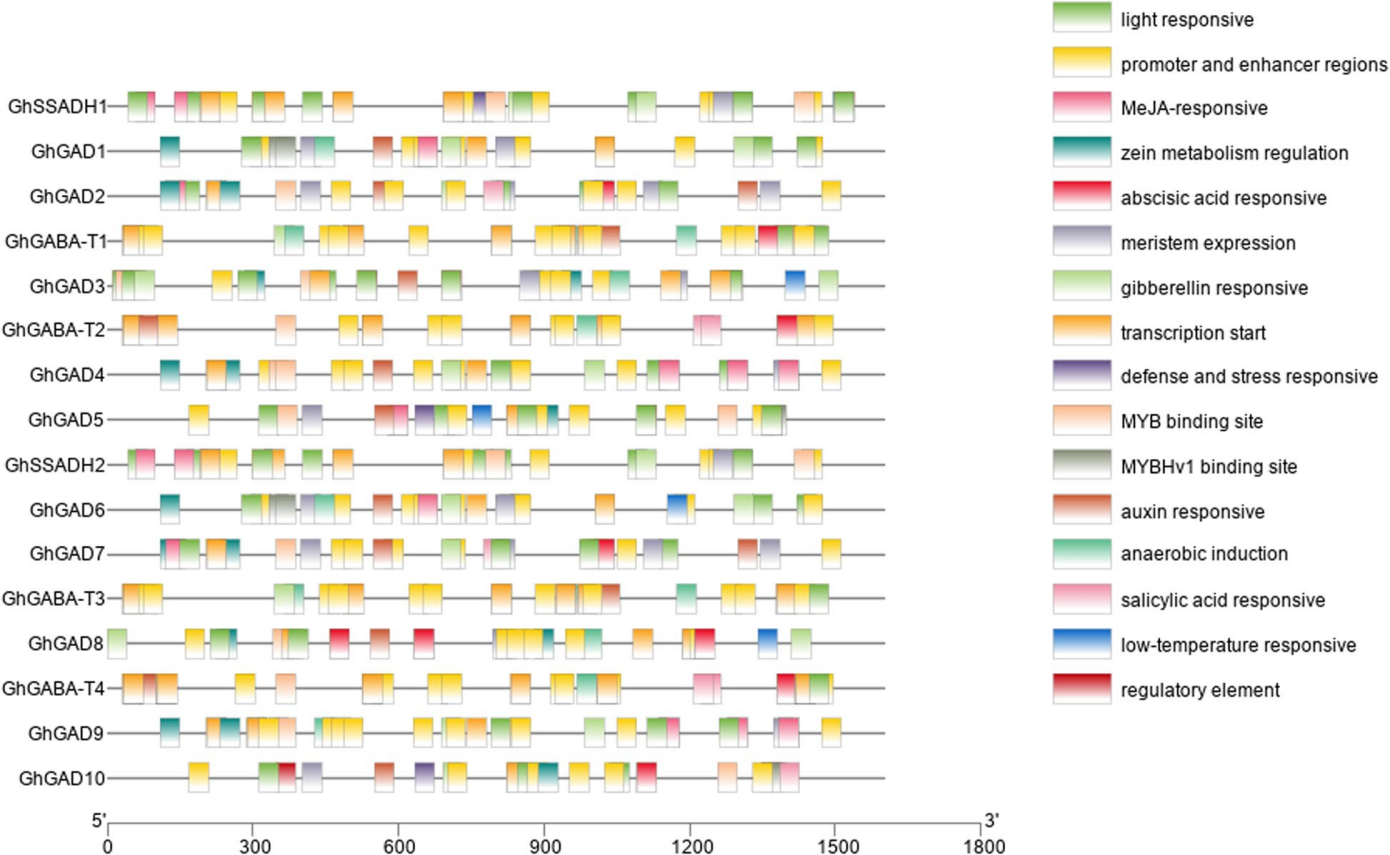
Promoter analysis of members of the three gene families of the *G. hirsutum* GABA branch

Promoter analysis showed that all members of the three gene families of the *G. hirsutum* GABA branch contained elements related to responses to light, hormones, and the environment (Fig. 6). The transcription core elements were the most abundant of all elements; the hormone response elements were mainly related to auxin, abscisic acid, and gibberellin, salicylic acid; and the environmental response elements were mainly involved in defense and stress-response elements and anaerobic induction, low-temperature response, and drought induction. All 10 GAD family gene members except GhGAD9 and GhGAD10 contained auxin-response-related elements, and GhGAD2, GhGAD7, GhGAD8, and GhGAD10 contained elements related to abscisic acid response.

Among the elements related to environmental response, members of the GAD family all contained elements related to anaerobic induction; GhGAD3, GhGAD5, GhGAD6, and GhGAD8 contained cold-related elements; and GhGAD5 and GhGAD10 contained stress-response elements. All four GABA-T family members contained elements related to light response and anaerobic induction, and all other members except GhGABA-T3 also contained elements related to abscisic acid. All SSADH family genes contained elements related to light, gibberellin, and abscisic acid responses, and GhSSADH1 also contained elements related to stress response.

#### Promoters of GABA branch gene family members in *G. barbadense*

**Fig. 7:**
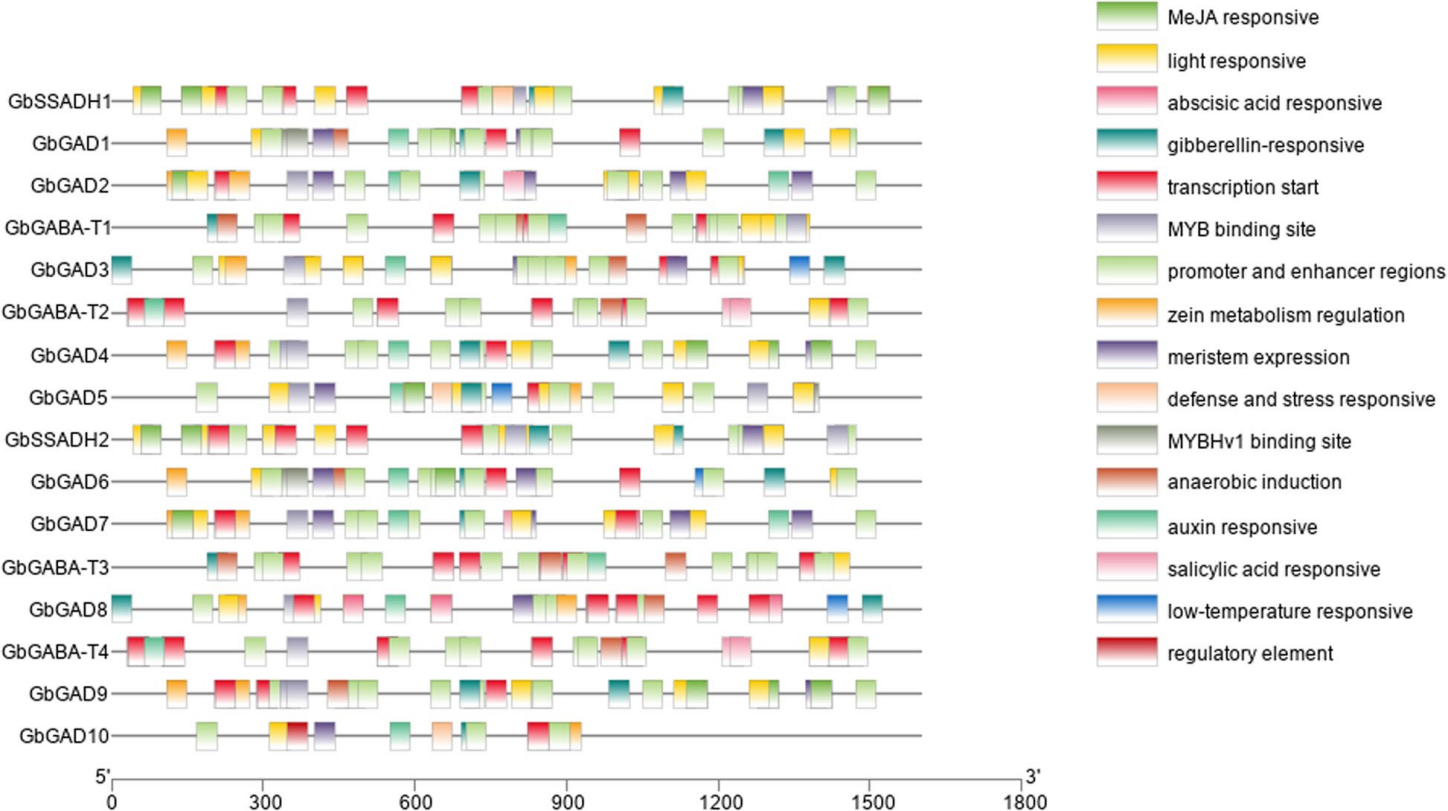
Promoter analysis of members of the three gene families in the *G. barbadense* GABA branch

Promoter analysis showed that all members of the three gene families of the GABA branch contained elements related to responses to light, hormones, and the environment (Fig. 7). The transcription core elements were the most abundant of all elements; the hormone response elements were mainly related to auxin, abscisic acid, gibberellin, and salicylic acid responses; and the environmental response elements were mainly involved in defense and stress-response elements and anaerobic induction, low-temperature response, and drought induction. All 10 of the GAD family gene members contained gibberellin- and auxin-responsive elements, and GbGAD2, GbGAD7, and GbGAD8 contained salicylic-acid-responsive elements.

Among the elements associated with environment-related responses, the GAD family gene members GbGAD1, GbGAD2, GbGAD3, GbGAD6, GbGAD8, and GbGAD9 contained elements associated with anaerobic induction, and GbGAD3, GbGAD5, GbGAD6, and GbGAD8 contained elements associated with low-temperature response. All four GABA-T family members contained elements related to light response and anaerobic induction, and all members except GbGABA-T3 also contained elements related to abscisic acid response. The SSADH family genes all contained light-responsive and gibberellin-responsive elements, and GbSSADH1 also contained abscisic-acid-responsive elements.

#### Promoters of GABA branch gene family in *G. Arboreum*

**Fig. 8:**
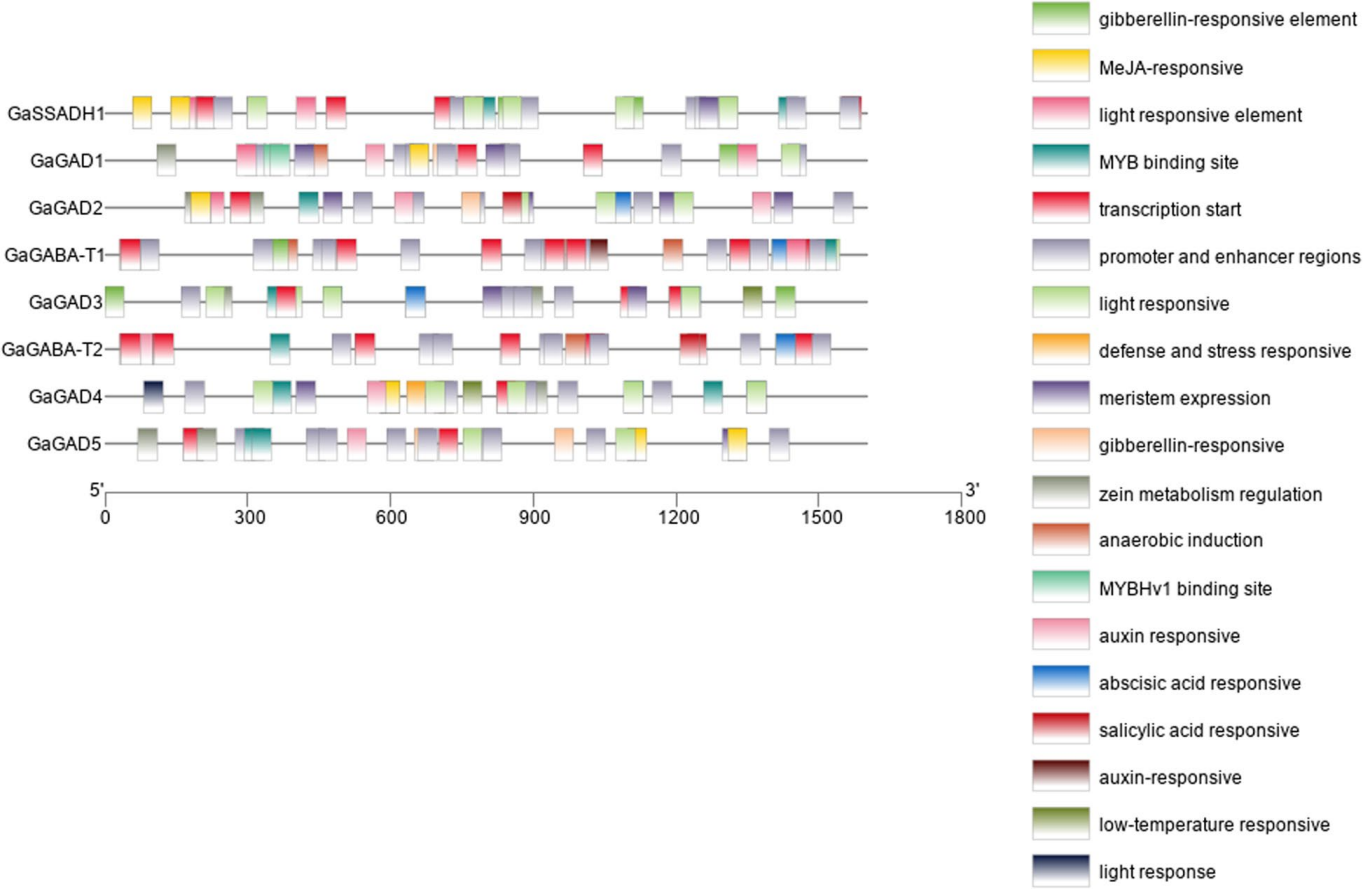
Promoter analysis of members of the three gene families of the *G. arboreum* GABA branch

Promoter analysis showed that members of the three gene families of the *G. arboreum* GABA branch contained elements related to light, hormones, and environmental responses (Fig. 8). Regulatory elements of promoters and enhancers had the largest number of all elements; hormone-response elements were mainly elements related to auxin, abscisic acid, gibberellin, and salicylic acid responses; and environmental response elements were mainly elements involved in defense and stress response and elements related to anaerobic induction, low-temperature response, and drought induction. In all five GAD family genes, GaGAD3 contained auxin-response elements, and GaGAD2 contained salicylic-acid-response elements.

Among the elements associated with context-related responses, the GAD family gene member GaGAD1 contained elements associated with anaerobic induction, and GaGAD3 and GaGAD4 contained elements associated with low-temperature response. Both GABA-T family members contained elements related to light response and anaerobic induction, and GaGABA-T1 contained gibberellin-responsive elements. One SSADH family gene contained light-responsive and gibberellin-responsive elements.

#### Promoters of GABA branch gene family in *G. Raimondii*

**Fig. 9:**
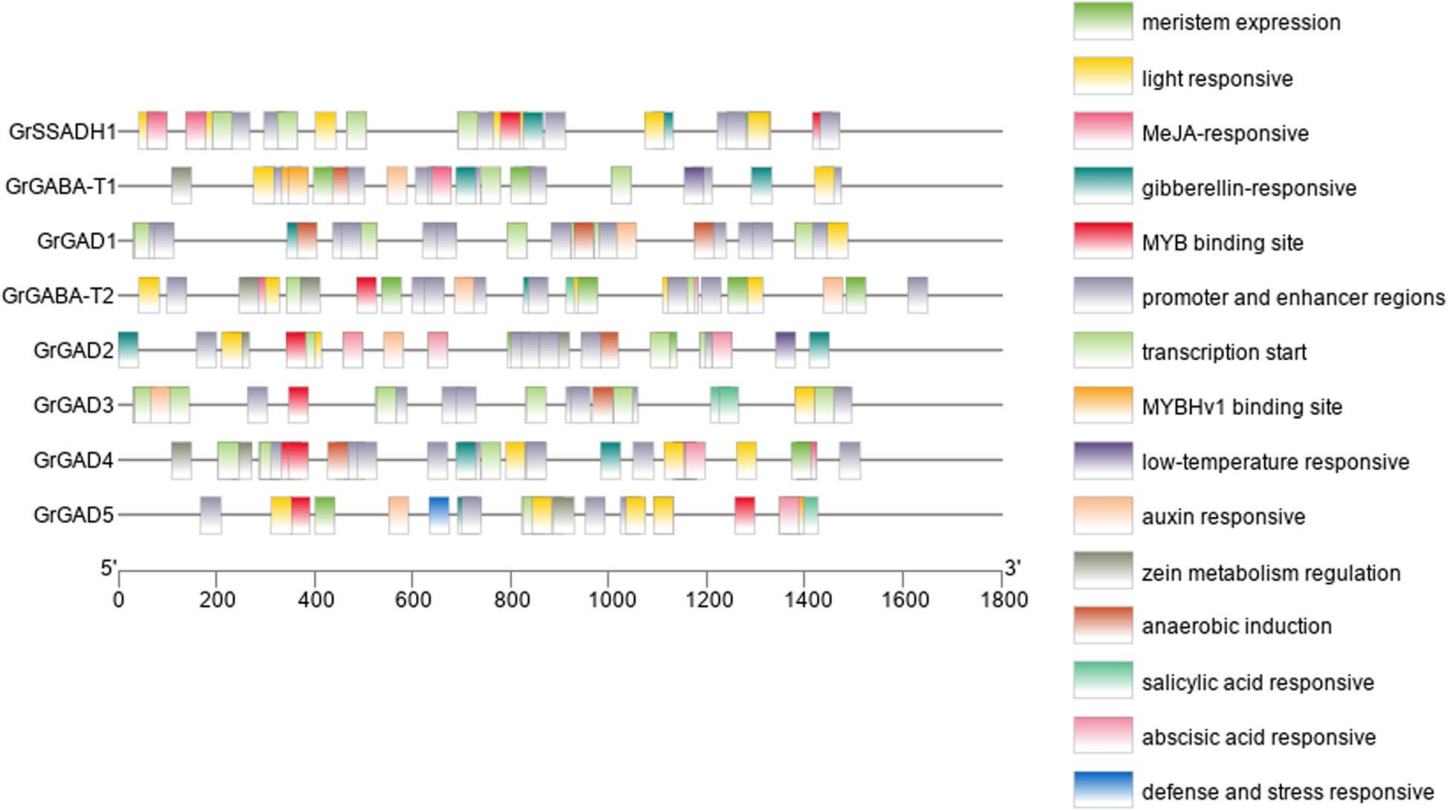
Promoter analysis of the *G. raimondii* GABA branch

Promoter analysis showed that members of the three gene families of the *G. raimondii* GABA branch contained elements related to responses to light, hormones, and the environment (Fig. 9). Regulatory elements of promoters and enhancers had the largest number of all elements; hormone-response elements were mainly elements related to auxin, abscisic acid, gibberellin, and salicylic acid responses; and environmental response elements were mainly involved in defense and stress response and elements related to anaerobic induction, low-temperature response, and drought induction. Among the five GAD family gene members, all but GrGAD3 contained gibberellin, and all except GrGAD4 also contained an auxin-responsive element and salicylic-acid-responsive elements.

Among the elements associated with context-related responses, the GAD family gene member GrGAD1 contained elements associated with anaerobic induction, and GrGAD2 contained elements associated with low-temperature response. Both GABA-T family members contained elements associated with light response, anaerobic induction, auxin, and salicylic acid, and GrGABA-T1 contained low-temperature-response-related elements. One SSADH family gene contained light-responsive and gibberellin-responsive elements.

#### Phylogenetic tree analysis of the GABA branch gene family members

**Fig. 10:**
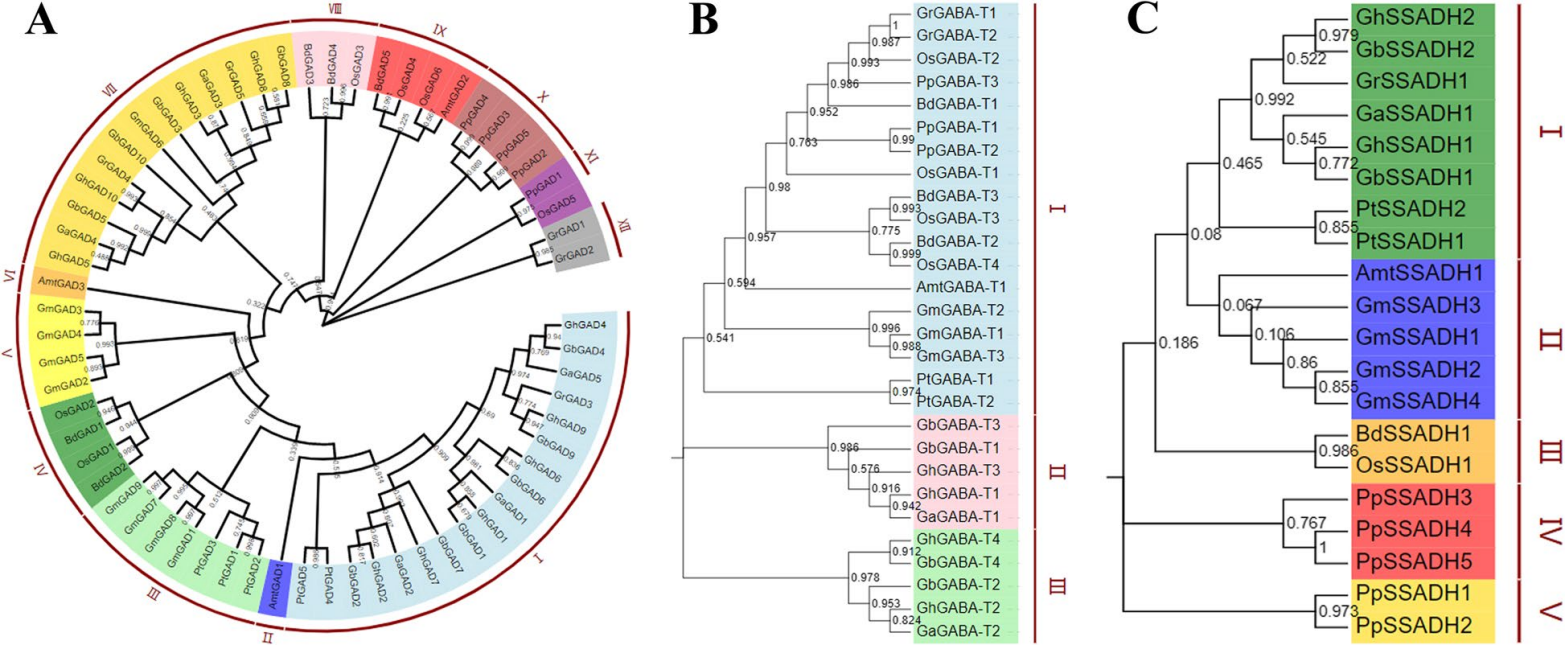
Phylogenetic tree of the GABA gene families. **A** Cotton GAD gene family and other plant gene families. **B** Phylogenetic tree of cotton GABA-T gene family and other plant gene families. **C** Phylogenetic tree of the cotton SSADH gene family with other plant gene families

 GABA branch family phylogenetic tree analysis of 10 species, namely *G. hirsutum*, *G. barbadense*, *G. arboreum*, *G. raimondii*, small Lithuanian moss, oil camphor, rice, soybean, two-spike short stalk, and poplar, found that *G. hirsutum*, *G. barbadense*, *G. arboreum*, and *G. raimondii* gene family members were gathered on a single branch (Fig. 10). GABA branch phylogenetic analysis found that the corresponding members of the three gene families in *G. hirsutum*, *G. barbadense*, *G. arboreum*, *G. raimondii*, and poplar clustered into one branch containing directly homologous genes, which further proved that the two originated from a common ancestor. GAD gene is the key gene in GABA synthesis, which was divided into 12 clusters among the 10 selected species (cluster); the cotton species and poplar were in Cluster I (Fig. 10A). The GAD genes of the cotton species were grouped into Clusters I and II, with GrGAD1 and GrGAD2 of *G. raimondii* in separate clusters. Thirty members of the GAD gene family of each cotton species were distributed in three different clusters, indicating homologous sequences in the family even within the same species. Quantitative analysis also found great differences in the roots, stems, and leaves. This suggests that different members of the GAD family have different functions in various parts of the cotton plant. GABA-T is a key gene of GABA degradation and metabolism, and among the selected 10 species, the members of this gene family were divided into three clusters (Fig. 10B). The GABA-T genes of all cotton species were clustered together, but the monocot rice had four GABA-T members, and the dicot *G. raimondii* and poplar had two, indicating that the family had lost genes in the process of dicotyledon evolution. The SSADH family members were divided into five clusters across the 10 selected species (Fig. 10C). The SSADH genes of each cotton species were divided, and *G. hirsutum*, *G. barbadense*, *G. arboreum*, and *G. raimondii* were closely related to the dicot plants.

Small Lithuanian moss, *Physomitrella patens*: PpGAD1. Pp3c2_9290V3.1; PpGAD2. Pp3c8_7810V3.1; PpGAD3. Pp3c14_21530V3.1; PpGAD4. Pp3c17_ 22200V3.1; PpGAD5. Pp3c23_8800V3.1; PpGABA-T1. Pp3c1_32910V3.1; PpGABA-T2. Pp3c6_25350V3.1, PpGABA-T3. Pp3s30_720V3.1; PpSSADH1. Pp3c1_16240V3.1; PpSSADH2. Pp3c6_25370V3.1; PpSSADH3. Pp3c6_27580V3.1; PpSSADH4. Pp3c26_10958V3.1; PpSSADH5. Pp3c26_ 10950V3.1.

Soybean, *Clycine max*: GmGAD1. Glyma.02G241400. 1; GmGAD2. Glyma.05G136100. 1; GmGAD3. Glyma.08G091300. 1; GmGAD4. Glyma.08G091400.1; GmGAD5. Glyma.08G091500.1; GmGAD6. Glyma.09G168900.1; GmGAD7. Glyma.11G213000. 1; GmGAD8. Glyma.14G211100.1; GmGAD9. Glyma.18G043600.1; GmGABA-T1. Glyma.03G057900.1; GmGABA-T2. Glyma.11G096200.1; GmGABA-T3. Glyma.12G022300.1; GmSSADH1. Glyma.03G052700. 1; GmSSADH2. Glyma.08G163700. 1; GmSSADH3. Glyma.14G091500. 1; GmSSADH4. Glyma.15G263500.1.

Brachypodium, *Brachypodium distallyan*: BdGAD1. Bradi1g10480.1; BdGAD2. Bradi1g68807.2; BdGAD3. Bradi3g37830.1; BdGAD4. Bradi5g11600.1; BdGAD5. Bradi5g11640.1; BdGABA-T1. Bradi2g31220.1; BdGABA-T2. Bradi3g17762.2; BdGABA-T3. Bra⁃ di5g21710.1; BdSSADH1. Bradg05490.1.

Rice, *Oryza sativa*: OsGAD1. LOC_Os03g13300.1; OsGAD2. LOC_Os03g51080.1; OsGAD3. LOC_Os04g37460.1; OsGAD4. LOC_Os04g37500.1; OsGAD5. LOC_Os05g34840.1; OsGAD6. LOC_Os08g36320.1; OsGABA-T1. LOC_Os02g02210.1; OsGABA-T2. LOC_Os04g52440.1; OsGABA-T3. LOC_Os04g52450.1; OsGABA-T4. LOC_Os08g10510.1; OsSSADH. LOC _ Os02g07760.1.

Oil camphor, *Amborella trichopoda*: AmtGAD1. evm_27.model.AmTr_v1.0_scaffold00024.92, AmtGAD2. evm_27.model.AmTr_v1.0_scaffold00024.94; AmtGAD3. evm_27.model.AmTr_v1.0_scaffold00024.104; AmtGABA-T1. evm_27.model.AmTr_v1.0_scaffold00062.203; AmtSSADH.evm_27.model. AmTr_v1.0_scaffold00004.211.

Mao Guo Yang, *Populus trichcarpa*: PtGAD1. Potri.T059200.1; PtGAD2. Potri.004G075200.1; PtGAD3. Potri.004G075300.1; Pt⁃GAD4. Potri.010G100500.1; PtGAD5. Potri.008G141100.1, PtGAD6. Potri.012G039000.1; PtGABA-T1. Potri.016G018500.1; PtGABA-T2. Po⁃tri.006G020900.1; PtSSADH1. Potri.010G174000.1; PtSSADH2. Potri.008G081900.1.

### GABA gene family distribution on the chromosomes

**Fig. 11:**
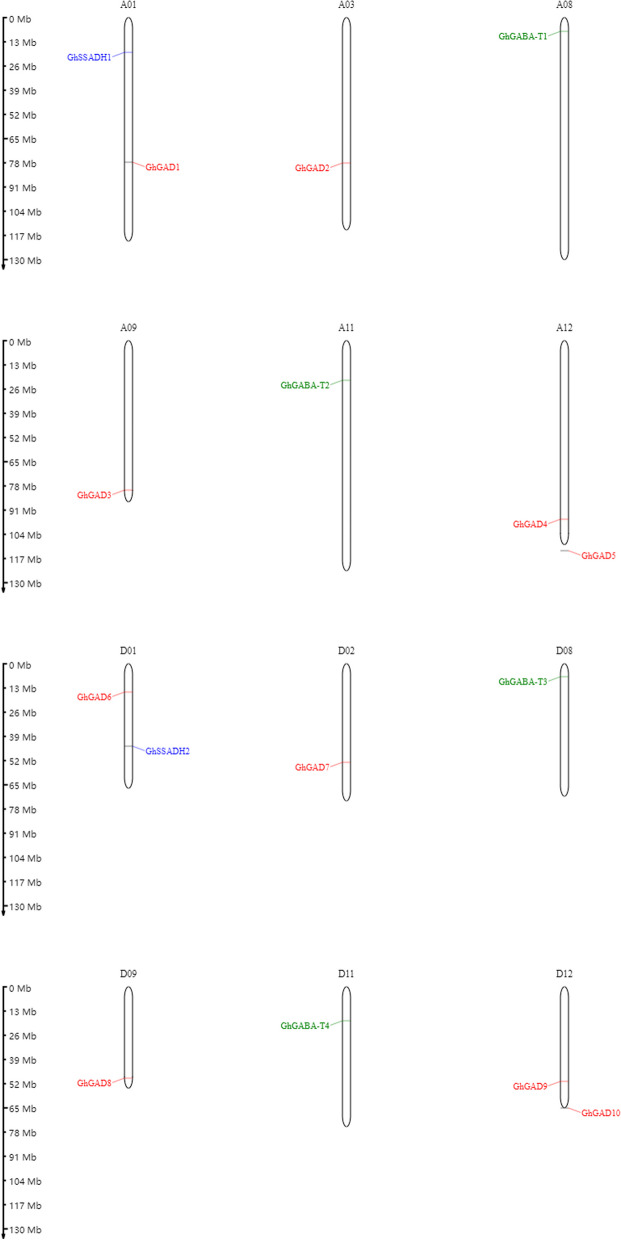
Location of the GABA branch genes on the chromosomes

**Fig. 12:**
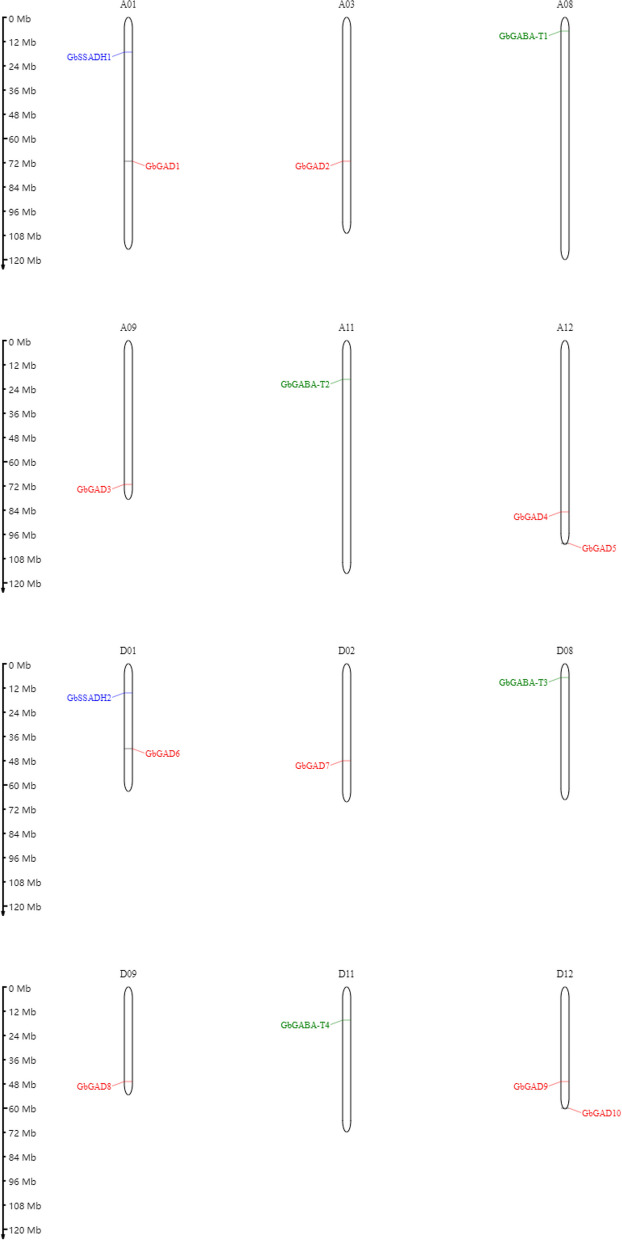
Location of the GABA branch genes on the chromosomes

**Fig. 13:**
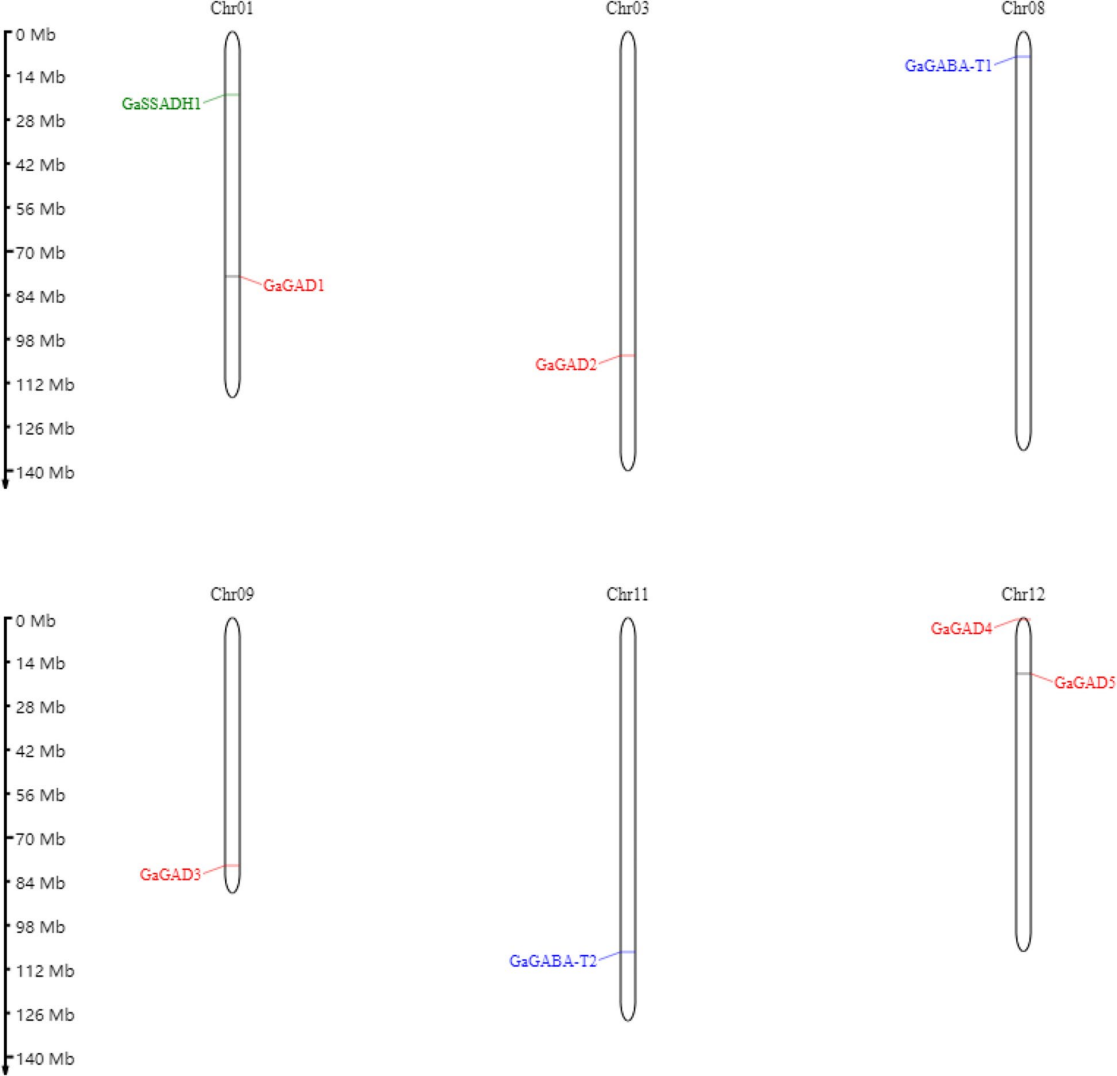
Location of the GABA branch genes on the chromosomes

**Fig. 14:**
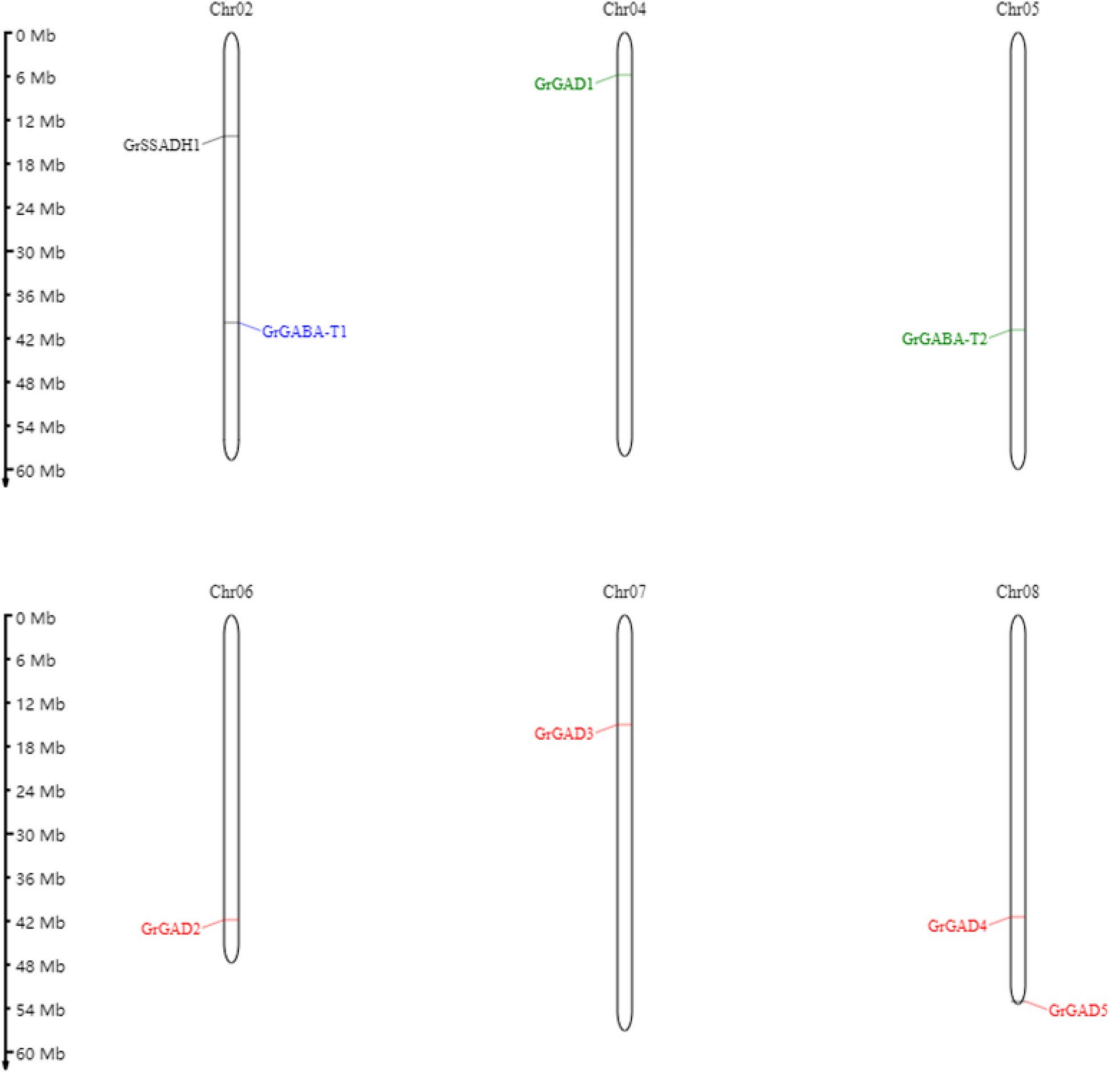
The position of the *G. raimondii* GABA branch genes on the chromosomes

The location information of genes on chromosomes provides an important reference for the evolution and function of gene families. This experiment revealed the position information of GAD, GABA-T, and SSADH genes on the chromosomes (Figs. 11, 12, 13 and 14).

#### Distribution of GABA gene family members on the chromosomes

The 16 genes involved in the GABA gene family were located on 12 chromosomes, with 8 in group A and 8 in group D. The distribution of the 16 screened genes was assessed across 12 chromosomes, namely chromosomes A01, A12, D01, and D12; and A03, A08, A09, A11, D02, D08, and D11, with one gene each. At the same time, the study found that the distribution on the chromosomes was not uniform in terms of the number of genes on each chromosome, and the gene distribution within each chromosome was also uneven. Groups A and D contained a symmetrical distribution of gene number and position, for example with A01 and D01 containing two genes with the same distribution positions in the upper and lower chromosome. A12 and D12 contained two genes each with the distribution position at the bottom of the chromosome. A08 and D08 contained one gene each with both distribution positions at the top of the chromosome. A09 and D09 contained one gene each with the distribution positions at the bottom of the chromosome. A11 and D11 contained one gene each with the distribution positions in the middle of the chromosome. A03 contained one gene in the lower middle position. D02 contained one gene in the lower middle position (Fig. 11).

#### Distribution of sea island Cotton GABA gene families on the chromosome

The 16 screened genes related to the GABA gene family were located on 12 chromosomes, 8 in group A and 8 in group D. The distribution of the 16 screened genes was assessed across 12 chromosomes, namely chromosomes A01, A12, D01, and D12; and A03, A08, A09, A11, D02, D08, and D11, with one gene each. At the same time, the study found that the distribution on the chromosomes was not uniform in terms of the number of genes on each chromosome, and the gene distribution within each chromosome was also uneven. Groups A and D contained a symmetrical distribution of gene number and position, for example with A01 and D01 containing two genes with the same distribution positions in the upper and lower chromosome. A12 and D12 contained two genes each with distribution positions at the bottom of the chromosome. A08 and D08 contained one gene each with both distribution positions at the top of the chromosome. A09 and D09 contained one gene each with both distribution positions at the bottom of the chromosome. A11 and D11 contained one gene each with both distribution positions in the middle of the chromosome. A03 contained one gene in the lower middle position. D02 contained one gene in the lower middle position. Unlike *G. hirsutum*, the SSADH gene was located in the upper middle (Fig. 12).

#### Distribution of the GABA gene family members on the chromosome in Asian cotton

The eight screened *G. arboreum* genes involved in the GABA gene family were located on six *G. arboreum* chromosomes. The distribution of eight genes was uneven, with chromosomes 1 and 12 containing two genes each, and chromosomes 3, 8, 9, and 11 containing one gene. At the same time, the *G. arboreum* GABA gene family distribution on the chromosome was not only uneven in terms of chromosome distribution but also in gene positions on the chromosomes being unevenly distributed, such as chromosome 1 with genes in the middle and lower chromosome, chromosome 3 with a gene in the lower chromosome, chromosome 8 with a gene in the upper chromosome, chromosome 9 and chromosome 11 with genes in the middle and lower chromosome, and chromosome 12 with genes at the top and in the upper chromosome (Fig. 13).

#### Distribution of the GABA gene family on the chromosomes in Raymond’s cotton

The eight related screened genes in the GABA gene family were located on six chromosomes in *G. raimondii*. The distribution of the eight genes was uneven, with chromosomes 1 and 8 containing two genes each, and chromosomes 4, 5, 6, and 7 containing one gene each. At the same time, the distribution of the GABA gene family on the chromosomes in *G. raimondii* was uneven not only in terms of the number of genes on each chromosome, but also in the gene positions on the chromosomes being unevenly distributed, such as chromosome 1 with genes in the upper and lower chromosome, chromosome 4 and chromosome 5 with a gene in the lower chromosome, chromosome 6 with a gene in the lower chromosome, chromosome 7 with a gene in the upper chromosome, and chromosome 8 with genes in the lower chromosome (Fig. 14).

### Analysis of gene duplication events of the GABA Family in G. Hirsutum

**Fig. 15:**
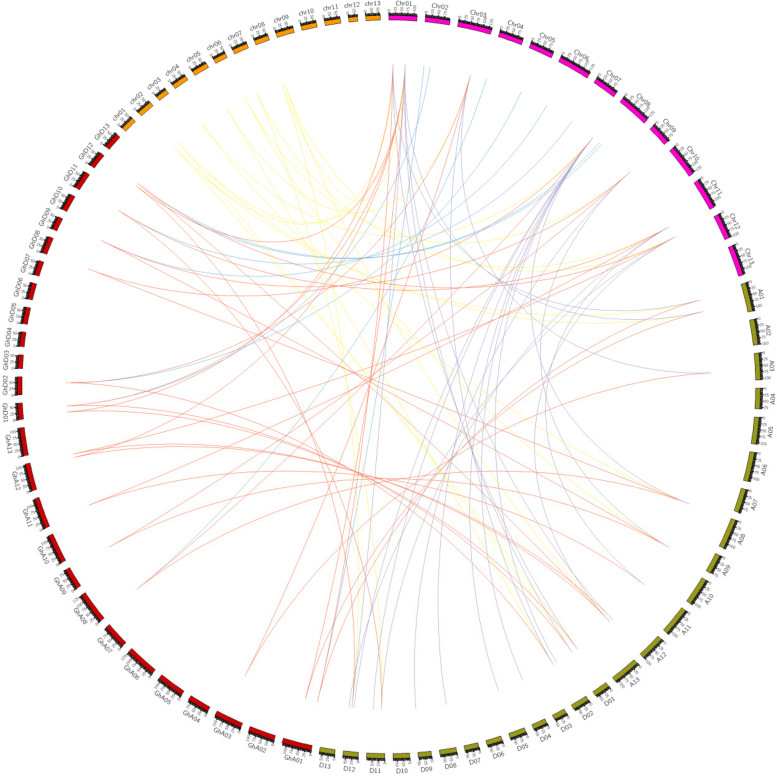
Collinearity analysis of cotton genes.(The chromosome id of Gossypium_hirsutum(UTX) was changed from A01 to GhA01. Change the chromosome id of Gossypium_raimondii(JGI), such as Chr01 to chr01, etc. Chromosome id of Gossypium_arboreum(CRI) : for example, Chr01; Chromosome id of Gossypium_barbadense(ZJU) : e.g. A01)

In this study, gene duplication events in the GABA gene family were assessed, including tandem and segmental duplications. Gene duplication plays an important role in the evolution of organisms, replicating genes to enable a plant to undergo physiological and morphological changes. This made it possible to explore the tandem duplication relationship among the cotton GABA family genes through sequence alignment screening. In the GABA gene family, 56 duplication events were found (Fig. 15). Combining the distribution of GABA gene family genes on the chromosomes revealed 56 pairs of duplication events as tandem duplication events, with all duplication events less than 200 kb apart.

In order to weigh the environmental selection pressures on these GABA gene families, the nonsynonymous substitution rate (Ka) and the synonymous substitution rate (Ks) of the replicated genes were calculated, and their ratios (Ka/Ks) were calculated for analysis. As shown in the table, the Ka/Ks ratios of the GABA gene family genes were much lower than 1, indicating that the GABA gene family genes have evolved under purifying selection, and that the functions of these genes are not seriously differentiated (Table 6).

**Table 6:** Analysis of evolutionary selection pressure on GABA family genes in *G. hirsutum*

Gene 1	Gene 2	Nonsynonymous Mutation Rate Ka	Synonymous Mutation Rate Ks	Ka/Ks
Gohir.A01G144400.v2.1	Gohir.A12G153200.v2.1	0.030369	0.588214	0.05163
Gohir.D09G168600.v2.1	Gohir.A01G100200.v2.1	0.878042	1.714269	0.50384
Gohir.D02G138300.v2.1	Gohir.A03G113900.v2.1	0.001726	0.027221	0.063394
Gohir.D08G060900.v2.1	Gohir.A08G051200.v2.1	0.006099	0.055758	0.109382
Gohir.A12G153200.v2.1	Gohir.D01G136200.v2.1	0.029463	0.59569	0.04946
Gohir.D12G156600.v2.1	Gohir.D02G138300.v2.1	0.035813	0.553538	0.064699
Gohir.D11G163100.v2.1	Gohir.D08G060900.v2.1	0.076391	0.381251	0.200371

### Protein interaction networks and functional annotations

**Fig. 16:**
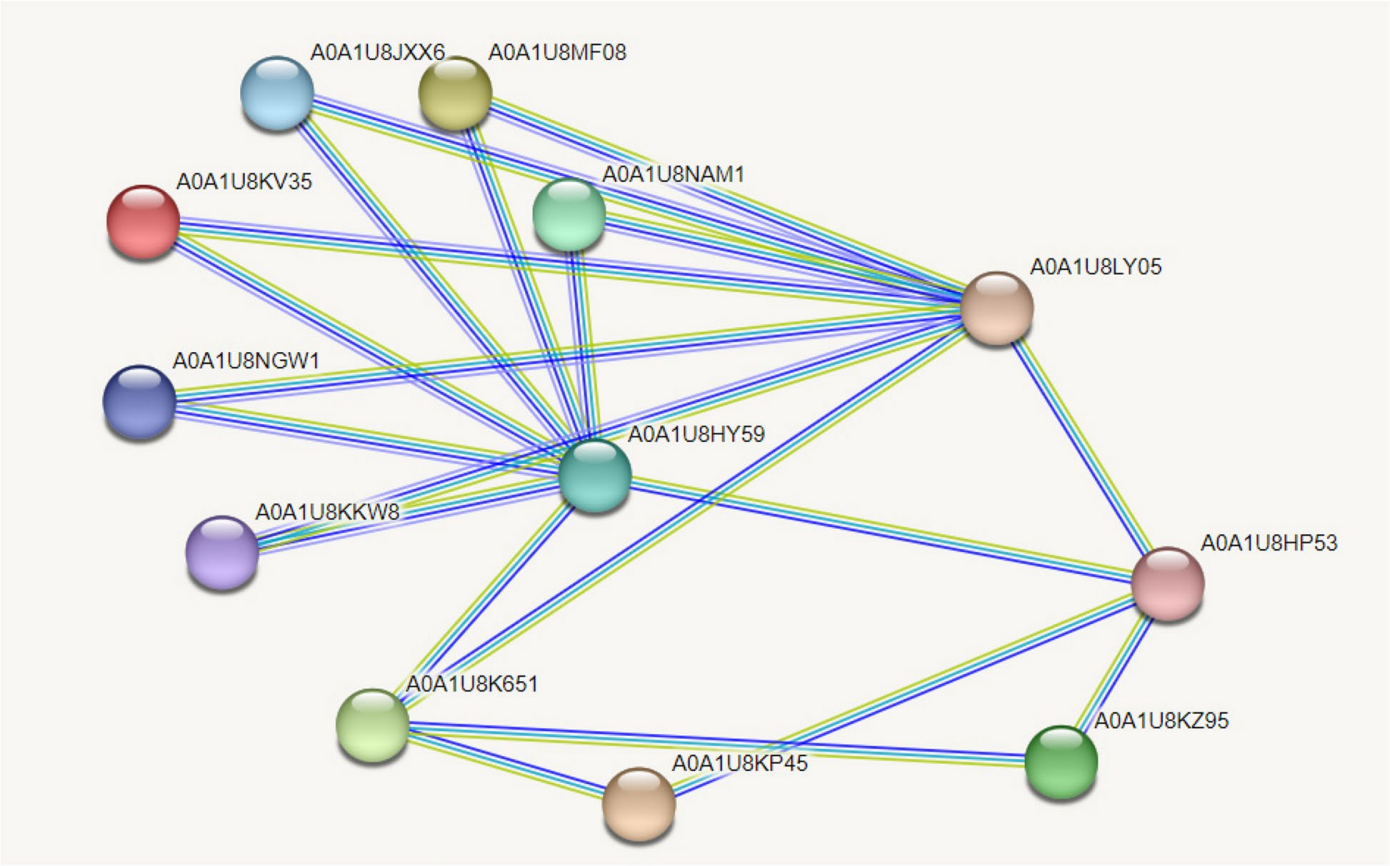
Protein–protein association network of the GhGABA genes based on their available information. The online tool STRING was used to predict the entire network. Different line colors represent the types of evidence for the associations, which are shown in the legend

Multiple proteins can form homodimers or heterodimers that bind to DNA and regulate the transcription process of their targets; hence, protein–protein interactions are of fundamental importance in gene expression. The differentially expressed protein interaction network was built using the default settings, except for the organism, confidence (score), and no more than 10 interactors. The amino acid sequences of the GABAs were input against the database, which contained all known and predicted protein–protein interactions. Among the identified GhGABA proteins, only 10 showed co-expression, while many GhGABA proteins were linked through direct interaction and showed co-expression supported by high scores. Most of the interactions were also supported by the constructed phylogenetic tree. Thus, this profiling can aid in predicting the functions of the uncharacterized partners (Fig. 16).

### GO and KEGG analysis of GABA family genes

**Fig. 17:**
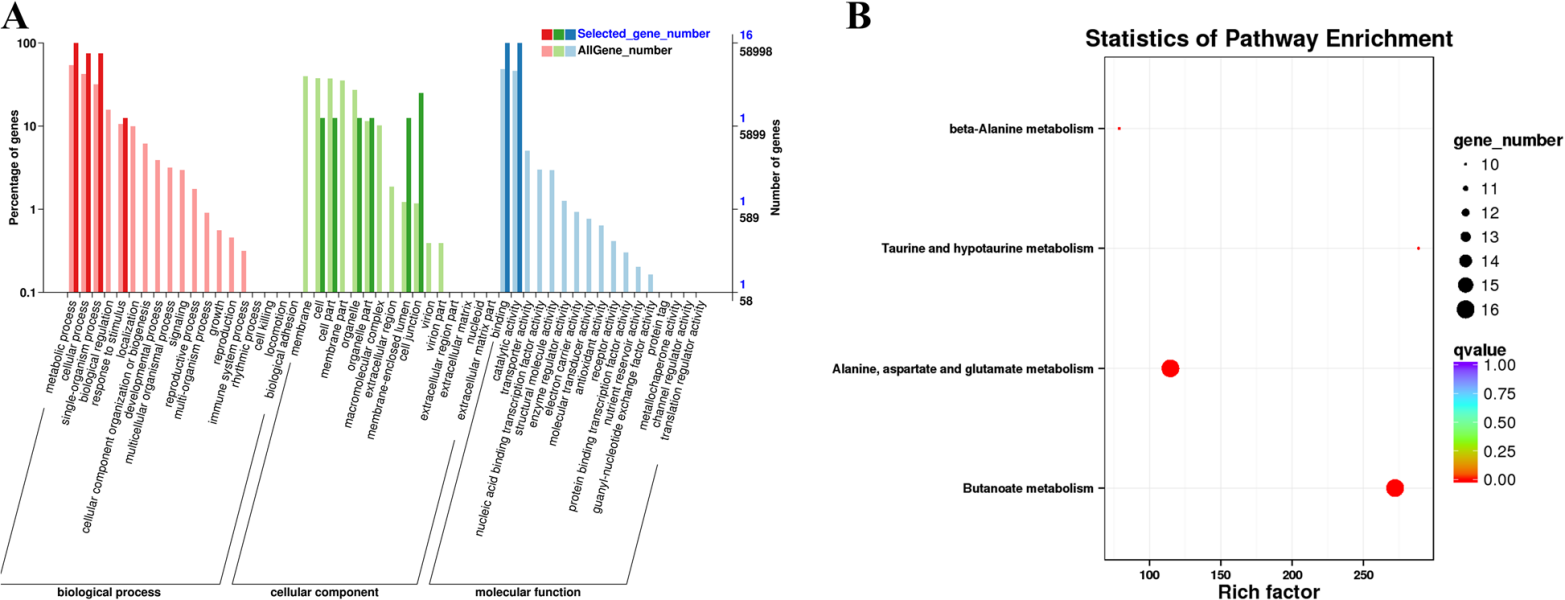
**A** Histogram of GO enriched genes. **B **Bar chart and bubble chart of KEGG enriched genes

**Fig. 18:**
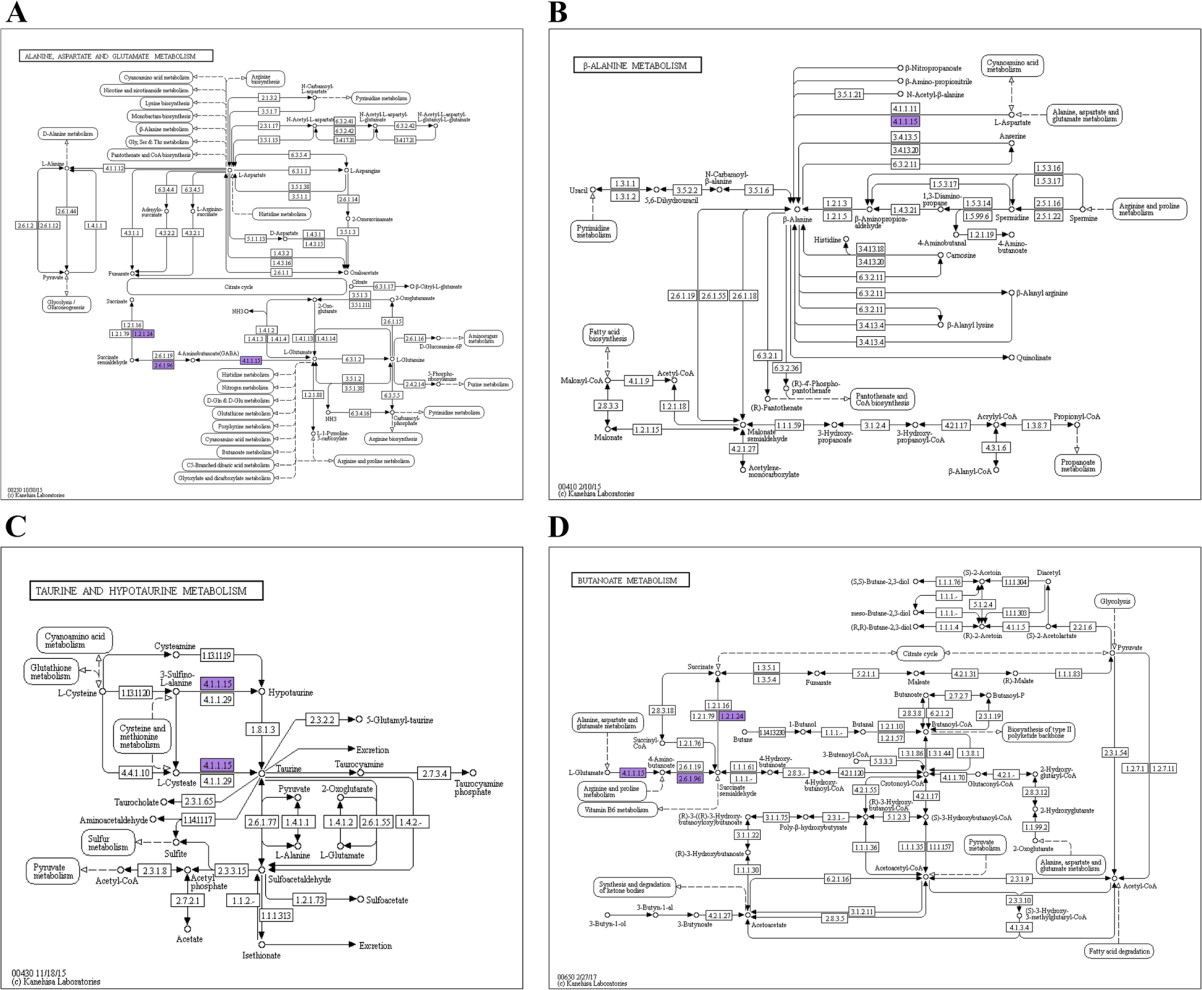
KEGG annotation path diagram. The numbers in the boxes represent the number of enzymes (EC number), and the entire pathway is composed of a complex biochemical reaction catalyzed by a variety of enzymes. In this pathway diagram, the enzymes related to differentially expressed genes are marked with different colors

Differential genes (DEGs) were enriched in multiple pathways with factors greater than 100, including alanine, aspartate, and glutamate metabolism, and in methyl butyrate metabolism with factors greater than 250. In the GO function enrichment pathway, the biological processes of “metabolism”, “cell process”, “single organ process”, “stimulus”, “cell component parts”, “membrane close the cavity”, “cell link”, molecular function category “combined”, and “catalytic activity” of selected genes vs. all genes were upregulated (Fig. 17). The co-expression trend analysis of the identified drought-resistance genes offered more knowledge of these genes’ expression trends across samples. The functions of two genes were discovered using GO and KEGG analyses (Fig. 18). KEGG analysis showed that differentially expressed genes were enriched in hormone signal transduction, carbon metabolism, and photosynthesis, suggesting that these metabolic pathways may play an important role in plant growth and development and stress resistance. Hormone interactions in plant hormone signaling pathways can be used as secondary signaling molecules to control the expression of downstream stress-related genes. Carbohydrates serve not only as an energy source but also as signaling molecules that regulate plant development and resilience to adversity.

### Differential gene expression analysis of GABA family genes under drought stress and high-temperature stress

As seen from the cluster heatmap of differentially expressed genes, the expression difference between the upregulated genes and downregulated genes was consistent with the trend of the sequencing results, indicating that the sequencing results were reliable.

**Fig. 19:**
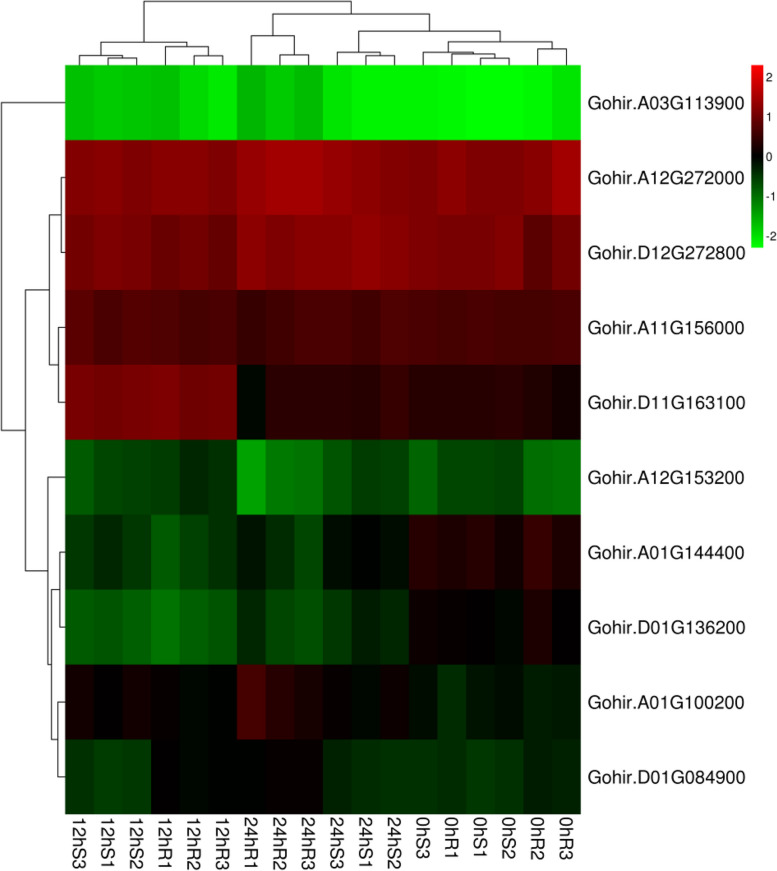
Heatmap of differentially expressed GABA family genes under drought stress

Figure 19 is a clustering map of differentially expressed genes; the columns represent different samples and the rows represent different genes. Clustering is based on log10 (FPKM + 1e-6) values. Red represents genes with high expression, and green represents genes with low expression. R_1_, R_2_, and R_3_ indicate the three replicates of the drought-resistant samples; S_1_, S_2_, and S_3_ indicate the three replicates of the drought-sensitive samples.

**Fig. 20:**
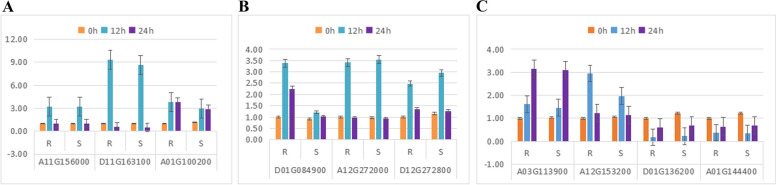
qRT-PCR validation of differentially expressed genes

Nine genes were chosen at random from the significantly differentially expressed genes for verification using real-time fluorescence quantitative PCR. The differences in expression between upregulated and downregulated genes were consistent with the trends of the sequencing data, showing that the sequencing results were trustworthy (Fig. 20).

**Fig. 21:**
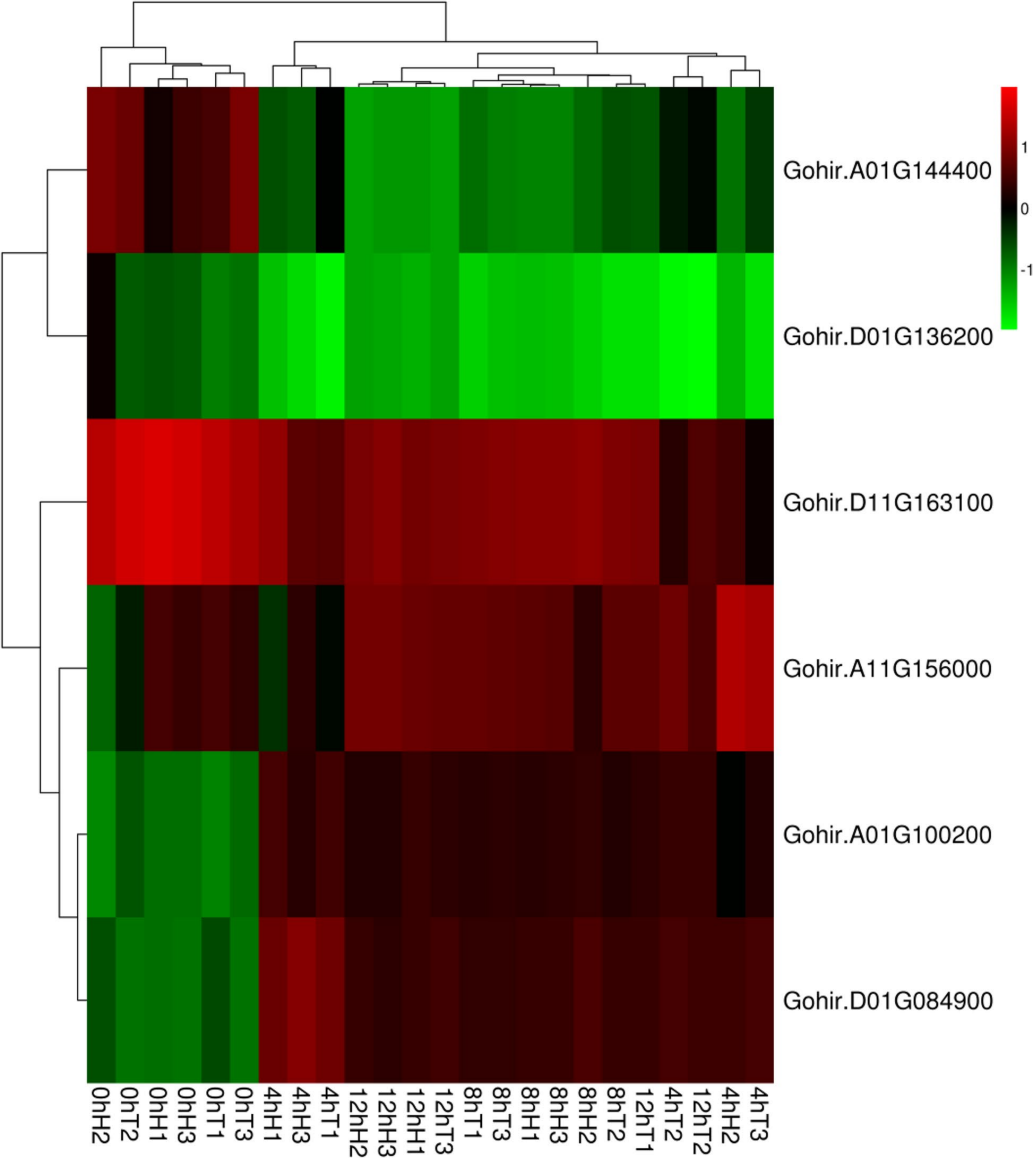
Cluster heatmap of differentially expressed GABA family genes under heat stress

Figure 21 is a clustering map of differentially expressed genes; the columns represent different samples and the rows represent different genes. Clustering is based on log10 (FPKM + 1e^−6^) values. Red represents genes with high expression, and green represents genes with low expression. H_1_, H_2_, and H_3_ represent the three replicates of the heat-resistant samples; T_1_, T_2_, and T_3_ represent the three replicates of the heat-sensitive samples.

**Fig. 22:**
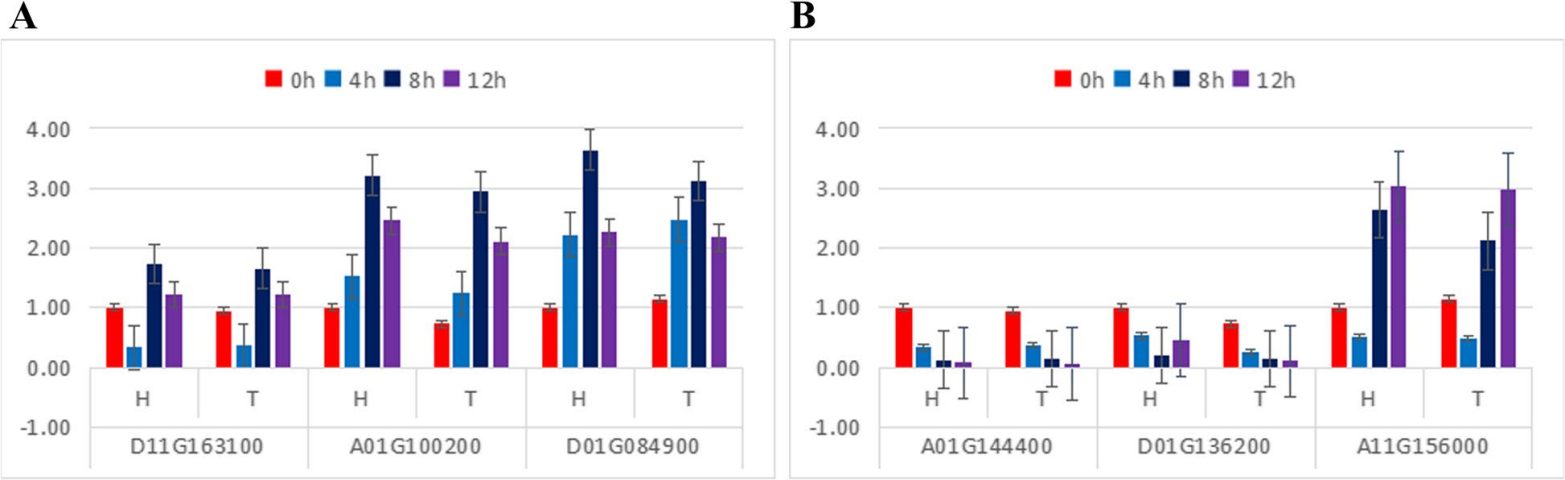
qRT-PCR validation of differentially expressed genes under heat stress

Six genes were chosen at random from the significantly differentially expressed genes for verification using real-time fluorescence quantitative PCR. The differences in expression between upregulated and downregulated genes were consistent with the trends of the sequencing data, showing that the sequencing results were trustworthy (Fig. 22).

### Whole-transcriptome ceRNA Network Analysis under Drought stress

**Fig. 23:**
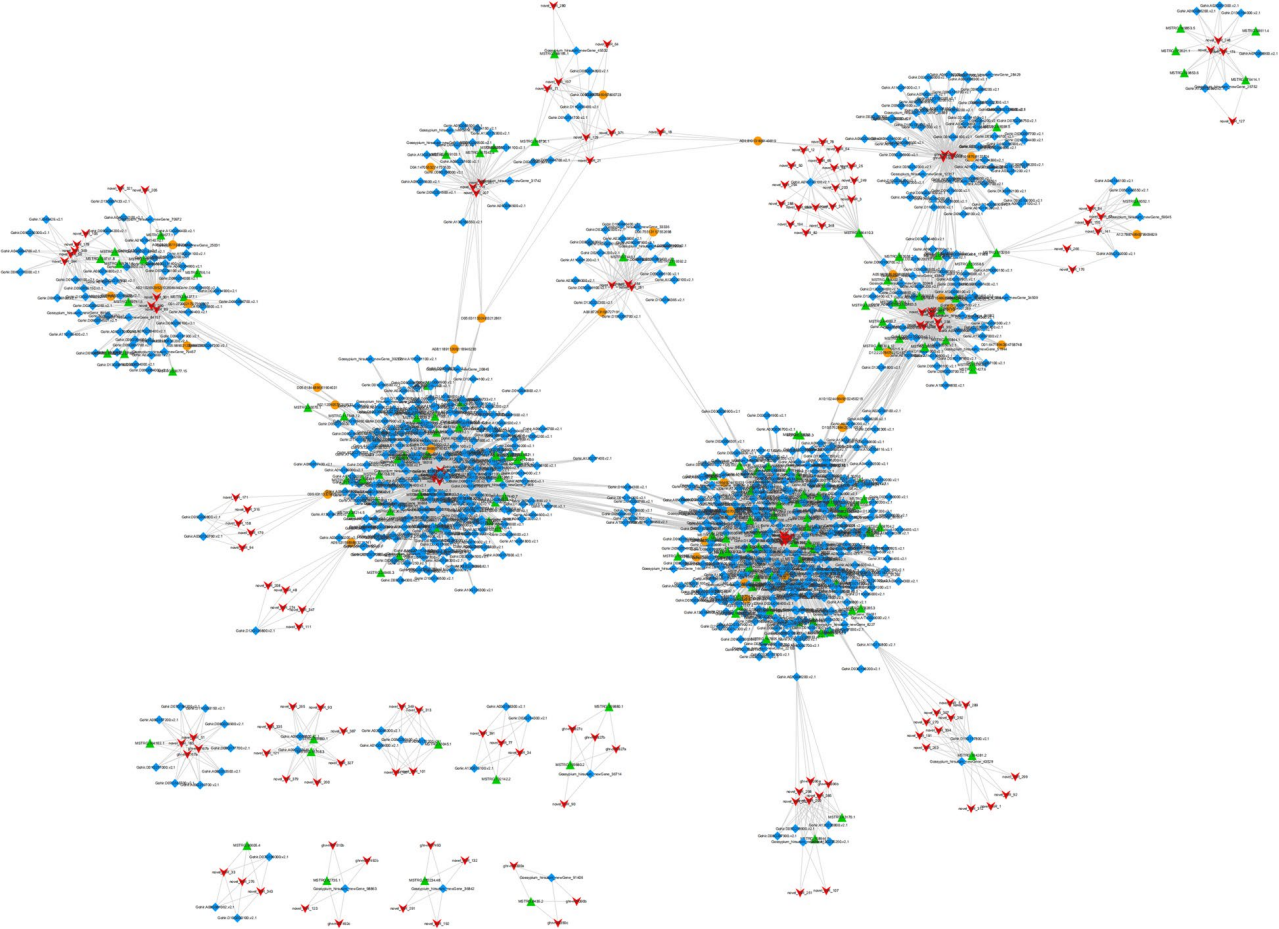
ceRNA regulation network. circRNA: orange circle. Gene: blue rhombus. lncRNA: upwards green triangle. miRNA: downwards red arrow

**Fig. 24:**
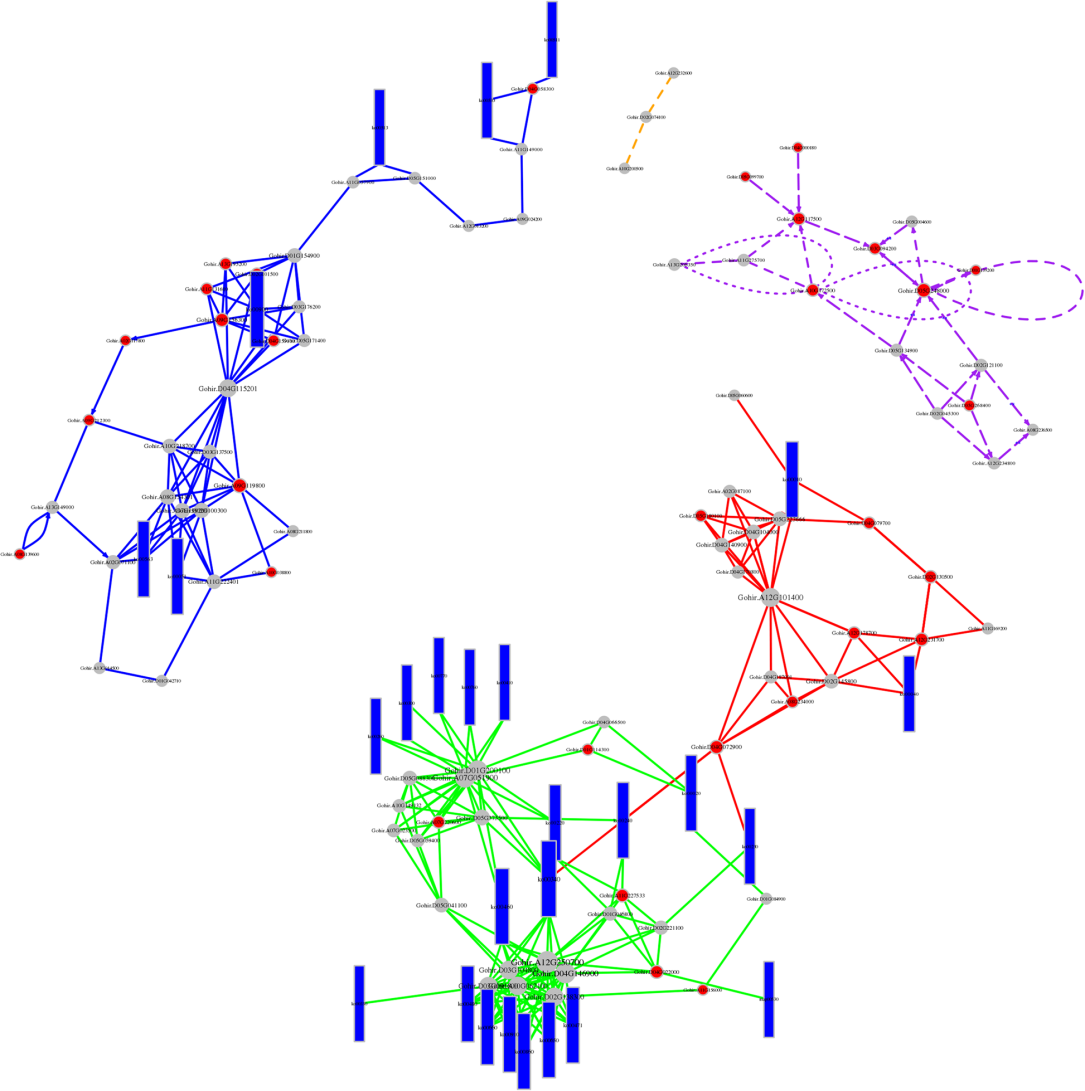
The top five most enriched pathways. Each dot represents a gene, each rectangle represents a pathway, and the line represents the relationship between genes and between genes and other pathways. The colors of different lines indicate that the relationship comes from different pathways. The red dots are the key genes

The whole drought-stress transcriptome was further analyzed to construct a ceRNA network. In this study, we used miRNA targeting to obtain candidate ceRNA relationships. A ceRNA relationship network was constructed using Cytoscape, and the ceRNA network contained 1289 edges and 1039 points, including 146 lncRNAs, 859 mRNAs, and 34 circRNAs (Fig. 23). Extracting each group of differentially expressed RNAs from the ceRNA relationship pair brings us one step closer to the differential ceRNA relationship pairs (Fig. 24). The classic algorithm PageRank in random walk was used to obtain the scores (that is, the importance) of all nodes in the network (that is, the different ceRNAs). A key RNA was selected as a key research object if it was ranked in the top 0.05 points in the network. Pathway enrichment analysis was performed on genes in key nodes + lncRNA target genes + circRNA host genes (hereinafter referred to as “key genes”), and the top five pathways with the most significant enrichment were selected. The relationships between all genes and genes in these five pathways were extracted and integrated into a pathway network, and key genes were mapped to pathways. Here, a node can be regarded as the ID of the ceRNA (gene/lncRNA/circRNA). Gohir.A11G156000 played an important role as a key gene in the alanine, aspartate, and glutamate metabolism pathways, which corresponded to the significant correlation between GABA content and glutamate, proline, and total free amino acid content, which was significantly different under drought, heat, and salt stress conditions with similar expression patterns, all reaching peak expression at 12 h. The Gohir.A11G156000 homologous gene was predicted to be the POP2 gene, encoding GABA aminotransferase. GABA-T catalyzes the conversion of GABA in the GABA shunt pathway to hemi-succinic acid (SSA), and some studies have shown that it is related to plant stress tolerance.

### Analysis of the results of exogenous GABA application

**Fig. 25:**
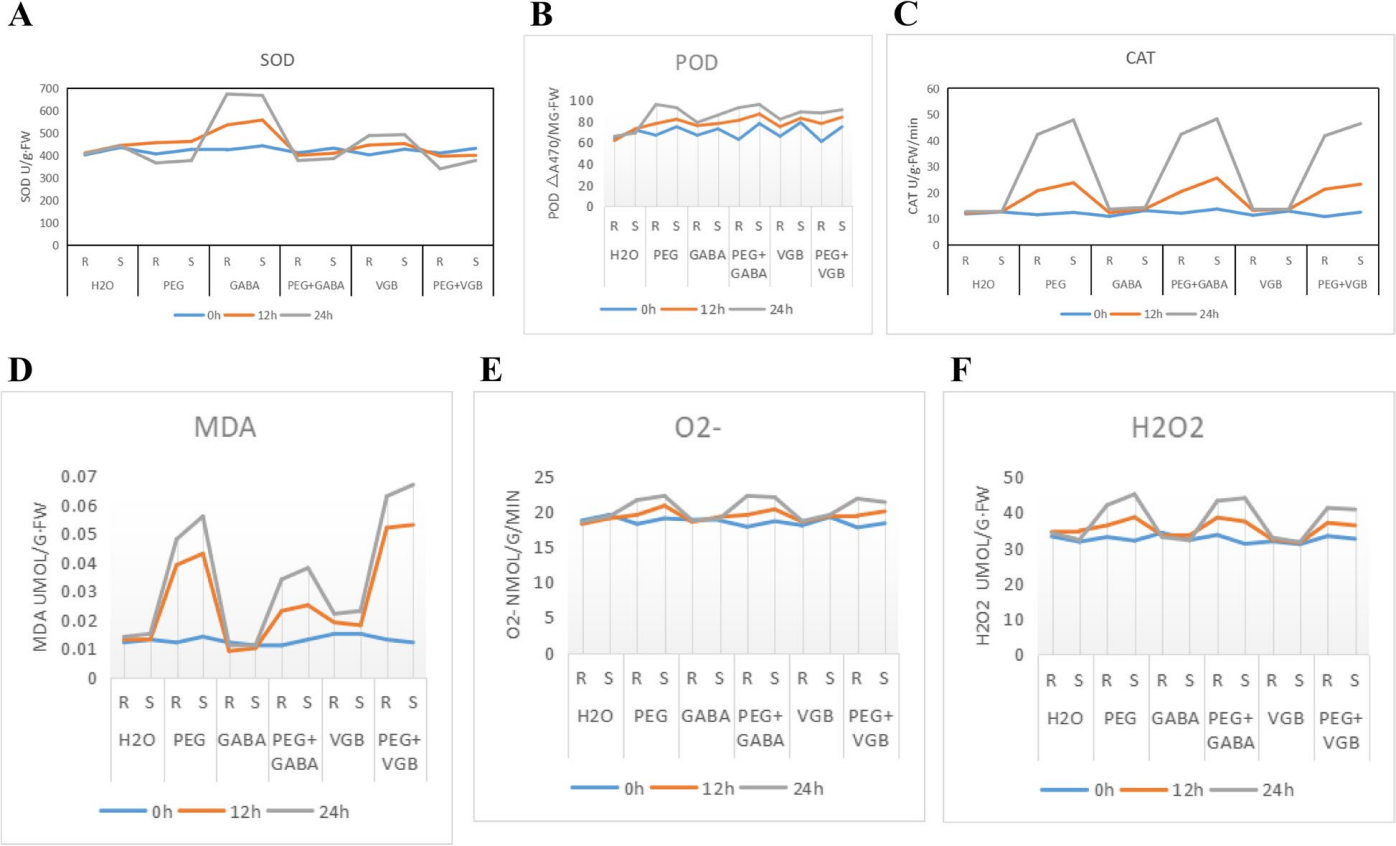
Responses to external GABA substrate application under drought conditions

This study showed that exogenous application of GABA can improve the activity of antioxidant enzymes SOD, POD, and CAT; reduce the rate of superoxide anion production and reduce the accumulation of hydrogen peroxide; delay the process of lipid peroxidation; and thus improve the stress tolerance of plants. Cao et al. found that exogenous GABA was able to significantly reduce endopeptidase activity in cotton leaves and roots. It was found that under salt stress, exogenous GABA treatment significantly promoted the elongation of seedling leaves and significantly increased the fresh weight of the shoot and lower parts, and the activities of leaf SOD, POD, and CAT were significantly increased, but the contents of ROS superoxide anion and MDA were significantly reduced. The above results showed that exogenous GABA partially attenuated abiotic stress damage by increasing leaf protective enzyme activity and reducing reactive oxygen species production. GABA was applied alone, and SOD activity was highest at 12 and 24 h (Fig. 25A), while the drought effect on POD activity was highest at 24 h (Fig. 25B). Results for PEG, PEG + GABA, PEG + VGB at 12 and 24 h and CAT, MDA, O^2−^, and H_2_O_2_ maximum activity are also shown (Fig. 25C,D,E,F).

**Fig. 26:**
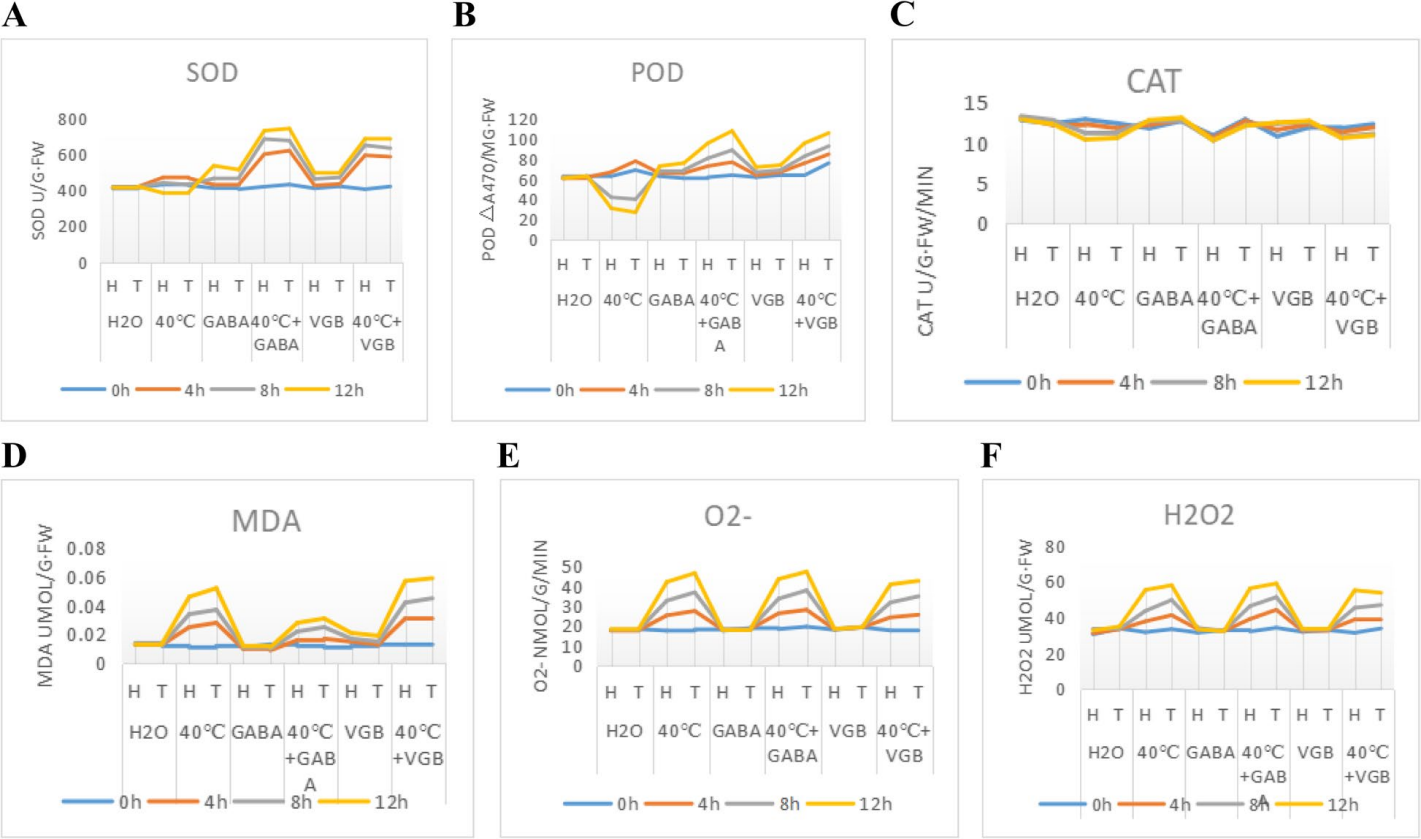
Effects of external GABA substrate application at high temperatures

Only GABA was most active at 4, 8, and 12 h (Fig. 26A).The effect of high temperature on POD activity was the highest at 12 h (Fig. 26B). Interestingly, there was no effect on CAT, MDA, O^2−^, or H_2_O_2,_ only on PEG, PEG + GABA, and PEG + VGB at 12 and 24 h (Fig. 26C,D,E,F).

### Analysis of GABA results in G. Hirsutum seedling field

In the Xinjiang cotton area from April to May, the unstable cold temperature, high salinity, low-lying cotton field area, duration of seedling formation, and the drought caused by water shortage can potentially lead to a decline in cotton seedling number, cotton yield, and cotton quality; prompt application of GABA can reduce these impacts on seedlings. The experiment was carried out over three consecutive years from 2019 to 2021 in the experimental site of Xinjiang Academy of Agricultural Sciences (80°50′31″ E, 40°30′13″ N). The experimental varieties were provided by the Cash Crop Research Institute of Xinjiang Institute of Agricultural Science and Technology. The tested varieties were the five *G. hirsutum* varieties most often cultivated locally (Table 7). A mechanized cotton planting system was utilized with drip irrigation under film and with six lines (66 + 10 cm). Each variety was planted in three replicates, in random arrangements of 3 m length and theoretical counts of 150,000 plants/mu. Field management was conducted together with field production.

**Table 7:** Seedling survival rate

Varieties	Year	Seedling Rate/%	
		H_2_O	GABA
Xinluzhong 80	2019	12.5	6.3
	2020	9.6	3.8
	2021	14.6	7.5
Xinluzhong 82	2019	12.6	8.3
	2020	9.8	6.1
	2021	7.1	5.9
Tahe 2	2019	18.2	11.3
	2020	16.7	9.6
	2021	13.2	6.3
Yuanmian 8	2019	6.8	3.2
	2020	7.3	2.5
	2021	8.6	4.3
Yuanmian 11	2019	9.3	4.6
	2020	7.7	2.5
	2021	8.4	3.7

## Discussion

Different subfamilies of the GABA branch gene family have diverse conserved protein motif distributions, which is the basis of the functional diversity of cotton and provides a reference for studying the functional differentiation between gene subfamilies in the same family. The distribution of the conserved protein motifs was generally consistent, indicating that the genes of the same subfamily have similar functions and close homologous relationships, which from another perspective proves the accuracy of the phylogenetic tree constructed in this study. Changes in GABA branch activity in plants can affect cellular pH, carbon and nitrogen balance, and hormone signaling pathways, so they play an important role in plant growth and development and defense against different stress mechanisms. Because promoters not only determine the transcription of genes but also regulate the level of gene expression, GABA branch (GAD, GABA-T, and SSADH 3) family genes were studied in upland, sea island, Asian, and *G. raimondii* cotton species. It was also found that each subfamily maintains a basic cotton gene number, 2:2:1:1, highlighting the evolutionary relationship among the three subfamilies.

Promoter analysis revealed that each family member contained elements related to its involvement in defense and stress, further demonstrating their function in protection against stress. Each family member also contained multiple elements related to responses to light, hormones, and the environment, which also proved their important roles in plant growth and development. In the GABA branch, GAD genes, as key genes catalyzing the synthesis of endogenous GABA, have been shown to perform important functions in plant growth and development and stress response.

Bo et al. focused on the gene Gohir.A11G156000. The subcellular localization showed its localization in mitochondria; the promoter expression was mainly in petals, stamens, stigma, and other organs, especially in stamens; and the expression activity in the stigma was stronger than in other sites. Yang found that the excessive expression of Gohir.A11G156000 reduced the GABA content and downregulated Gohir. The expression of A11G156000 increased GABA content and changed Gohir.A11G156000. The expression level in the cotton genome can affect the GABA metabolic pathway in cotton, thus changing the GABA content. The tolerance of wild-type plants to stress is significantly higher than that of knockout plants. The reason may be that the excessive expression of Gohir.A11G156000 increases the degradation level of GABA, thus accelerating the operating speed of the GABA branch. Under environmental stress conditions, the GABA branch can supply reaction substrates such as NADH and succinate to the TCA cycle, inhibit the accumulation of reactive oxygen species, and alleviate the toxicity caused by excessive oxygen under stress. GABA may have the same mechanism of action in other abiotic stresses, such as drought and heat stress, to improve cotton tolerance against stress, as demonstrated by the transcriptome results. Bo and other researchers found that the molecular patterns play critical roles in upregulating genes. The expression level of Gohir.A11G156000 increases the degradation of GABA, causing increased IAA content, increased chlorophyll content, enhanced photosynthesis rate, and the downregulation of Gohir. The expression level of Gohir.A11G156000 shows the opposite result, implying that the GABA content partly regulates the auxin accumulation in cotton roots. The production of low-molecular-weight osmotic adjustment substances such as GABA and other amino acids, polyols, and organic acids increased, and the expression of enzymes involved in antioxidant injury was upregulated [20]. GABA-T encoded by the Gohir.A11G156000 gene is the primary rate-limiting enzyme in GABA degradation and metabolism. GABA in plants is generated by decarboxylation of glutamate in the cytoplasm by GAD, and is then transported to mitochondria to produce succinate half-aldehyde through the catalysis of GABA-T. As a result, succinate is produced under the influence of SSADH, which is closely connected to the TCA cycle [5]. Studies show that GABA contains 50% of the total free amino acids in tomato. Arabidopsis can grow normally on medium with GABA as a nitrogen source, and, when plants are under stress, GABA degradation is required to produce succinate to provide a supplementary carbon source for the TCA cycle. It is concluded that GABA is closely linked to plant nitrogen and carbon metabolism.

Under normal growth conditions, the production and removal of reactive oxygen species in plant cells does not harm the cell; however, and multiple stress conditions trigger the accumulation of intracellular reactive oxygen species while disrupting their equilibrium. This leads to membrane peroxidation and deflipation, by which the cell structure and function are destroyed. The SOD, POD, CAT, and APX present in plant cells are important protective enzymes for free-radical scavenging and play a key role in combating oxidative stress. Many studies have shown that during the processes of stress and aging, cells produce H_2_O_2_ and O_2_ first-class reactive oxygen free radicals through various mechanisms, which leads to plant cell damage, such as degradation reactions, membrane lipid peroxidation, cell membrane degeneration, and protein degeneration. The presence of reactive oxygen species scavengers in plant cells can effectively remove reactive oxygen species such as H_2_O_2_ and O_2_, maintaining them at low levels and thus avoiding or alleviating membrane damage. Compared with controls, GABA treatment significantly increased the activities of SOD, POD, CAT, and APX and inhibited the overproduction of H_2_O_2_ and O_2_ under drought and heat stress, which indicates that GABA treatment could directly or indirectly improve antioxidant enzyme activity and reduce cell damage. Previous studies have shown that applying exogenous GABA can regulate the metabolism of reactive oxygen species in plants under abiotic stress, reduce the production rate of O_2_, alleviate the oxidative stress caused by excessive ROS accumulation, maintain the stability of the intracellular environment, protect the integrity of the chloroplast membrane structure, and maintain the normal growth and development of plants. Our results also showed that exogenous GABA alleviated damage to seedlings caused by drought and heat stress and reduced the accumulation of O_2_ and H_2_O_2_ in plants. concluded that the effect of GABA on the endogenous hormone content of white trifolium seedlings under drought stress showed that GABA could increase the endogenous content of endogenous abscisic acid and significantly increase the content of jasmonic acid, auxin, and isopentenyl adenine under drought stress, which could effectively alleviate the inhibition of plant growth by drought stress. GABA can improve the heat resistance of plants by improving the activity of superoxide dismutase and peroxidase in crops to protect tissue and organs under high-temperature stress. However, some studies have shown that the GABA content is not directly related to the stress tolerance of plants. Salt stress treatment destroyed the normal GABA metabolism in tomato plants and affected the metabolic balance of the downstream product succinate semialdehyde (SSA), thus reducing the supplementation of succinate to the TCA cycle. Knockdown of Sl GADs in tomato decreased the intracellular GABA concentration, whereas knockdown of SlGABA-Ts increased the intracellular GABA concentration, but both reduced the salt tolerance of tomato seedlings. Studies on Arabidopsis under salt stress have also found that disruption of the GABA breakdown process leads to a decrease in salt tolerance in plants, indicating that GABA catabolism is key to affecting the salt tolerance in Arabidopsis. Therefore, GABA content and salt tolerance are not directly related to this process, though the GABA shunt branch may play a key role in plant stress resistance. Studies have shown that GABA, in addition to being an intermediate of nitrogen metabolism, plays an important role in maintaining carbon and nitrogen balance. GABA also has strong hydrophilic and osmotic abilities, and under stress it has a similar function to proline: an osmotic regulation role. In addition, some studies have demonstrated that GABA may participate in intercellular signaling and play an important role in plant responses to stress. Therefore, the effects of GABA on plants and stress resistance may have multiple regulatory mechanisms. Through activity experiments on the GAD gene promoter, we found that in addition to NaCl-stress-induced activity, light, ABA, SA, and MEJA responses were also affected. On the one hand, this indicates that the expression of GAD genes is regulated by various factors, and it also suggests that GABA may participate in multiple regulatory pathways in plants.

In plants, GABA plays the dual role of a metabolic substance and a signaling substance, and is involved in plant pH regulation, energy metabolism, regulation of C/N balance, and defense system regulation. GABA can improve plant resistance to adverse conditions and has the role of promoting reproductive growth and vegetative growth. GABA is neither a pesticide nor a plant growth regulator, and it is not a traditional fertilizer but rather a green, safe, and efficient biological stimulator. It can be applied via multiple dilution and through roots, drip irrigation, and foliar spray applied to crops, and it can also be added to liquid, water-soluble, foliar, and compound fertilizers. It is suitable for indoor use and can also be added to pesticide. GABA is relatively stable in different pH conditions, is easily soluble in water, and has good recompatibility and compatibility with various media. It is believed that with the continuous popularization and application of biostimulators in agriculture, GABA, as a green and efficient product, will benefit agricultural workers and the agricultural industry.

## Conclusions

In the *G. hirsutum* genome, 151 genes of the GABA gene family were identified. Of these, 73 were on chromosomes of group A, 75 were on chromosomes of group D, and 3 others were distributed on three different scaffolds. In the *G. arboreum* genome, 85 genes of the GABA gene family were identified. Of these, 82 were mapped onto 13 chromosomes; the other 3 were distributed on three different scaffolds. In the *G. raimondii* genome, 86 genes of the GABA gene family were identified. Of these, 85 were located on 13 chromosomes, while the other gene was located on a scaffold. After phylogenetic analysis, the *G. hirsutum* GABA gene family was divided into 12 subfamilies, *G. arboreum* GABA genes into 10 subfamilies, and *G. raimondii* GABA genes into 11 subfamilies. The gene number ratio of the three cotton species in the systematic progressive development analysis was basically 2:1:1, which is consistent with the evolutionary relationship of the three cotton species.

Different genes in the upland, sea island, Asian, and *G. raimondii* cotton GABA gene families differed greatly in structure and conserved motifs of the encoding proteins but were very conserved in the same subfamilies. Large-scale segmental duplication is the main reason for the large number of GABA genes in *G. hirsutum*, and most of the *G. hirsutum* GABA duplication genes have undergone strong purifying selection in the process of evolution, and very few genes have been subjected to positive selection effects. The heatmap of the expression of GABA genes under salt stress indicates that the GABA gene responds to salt stress in *G. hirsutum* and participates in the regulation of expression. According to their expressions, 24 genes are speculated to have significant regulatory functions in response to salt stress.

## Data Availability

The raw sequence data presented in this research have been deposited in the National Center for Biotechnology Information (NCBI) under the accession numbers PRJNA769509 and PRJNA769837, and Accession No. PRJNA706603 are publicly accessible at https://www.ncbi.nlm.nih.gov/ (accessed on 1 January 2021).
